# Systematic literature review and meta-analysis of the prevalence of secondary progressive multiple sclerosis in the USA, Europe, Canada, Australia, and Brazil

**DOI:** 10.1186/s12883-022-02820-0

**Published:** 2022-08-17

**Authors:** Vijayalakshmi Vasanthaprasad, Vivek Khurana, Sreelatha Vadapalle, Jackie Palace, Nicholas Adlard

**Affiliations:** 1 Novartis Healthcare Pvt Ltd, Value & Access, NBS CONEXTS, Hyderabad, India; 2 Novartis Corporation (Malaysia) Sdn. Bhd, Kuala Lumpur, Selangor Malaysia; 3 Nuffield Department of Clinical Neurosciences, University of Oxford, Oxford, UK; 4 Novartis Pharma AG, Basel, Switzerland

**Keywords:** Meta-analysis, Multiple sclerosis, Prevalence, Secondary progressive multiple sclerosis

## Abstract

**Background:**

Secondary progressive multiple sclerosis (SPMS) is a subtype of multiple sclerosis (MS), which is a chronic neurological disease, characterised by inflammation of the central nervous system. Most of MS patients eventually progress to SPMS. This study estimates the prevalence of SPMS in the United States of America, Europe, Canada, Australia, and Brazil.

**Methods:**

A systematic literature search of the Medline and Embase databases was performed using the OVID™ SP platform to identify MS epidemiological studies published in English from database inception to September 22, 2020. Studies reporting the prevalence of MS and proportion of SPMS patients in the included population were selected. The pooled prevalence of SPMS was calculated based on the proportion of SPMS patients. The Loney quality assessment checklist was used for quality grading. A meta-analysis of the proportions was conducted in RStudio.

**Results:**

A total of 4754 articles were retrieved, and prevalence was calculated from 97 relevant studies. Overall, 86 medium- and high-quality studies were included in the meta-analysis. Most studies were conducted in European countries (84 studies). The estimated pooled prevalence of SPMS was 22.42 (99% confidence interval: 18.30, 26.95)/100,000. The prevalence of SPMS was more in the North European countries, highest in Sweden and lowest in Brazil. A decline in SPMS prevalence was observed since the availability of oral disease-modifying therapies. We also observed a regional variation of higher SPMS prevalence in urban areas compared with rural areas.

**Conclusion:**

High variability was observed in the estimated SPMS prevalence, and the quality of the studies conducted. The influence of latitude and other factors known to affect overall MS prevalence did not fully explain the wide range of inter-country and intra-country variability identified in the results.

**Supplementary Information:**

The online version contains supplementary material available at 10.1186/s12883-022-02820-0.

## Background

Multiple sclerosis (MS) has affected approximately 2.2 million people worldwide till 2016 [1]. MS epidemiological studies have consistently reported that 85% of MS patients start with relapsing-remitting MS (RRMS), of which the majority eventually develop secondary progressive MS (SPMS), often with superimposed relapses that tend to decline over time [2]. A systematic literature review of 92 studies reported that approximately 25% of patients with RRMS progress to SPMS by 10 years, 50% progress by 20 years, and over 75% progress by 30 years, with most studies reporting a mean age of 40 years at conversion to SPMS [3]. SPMS is usually diagnosed retrospectively by a history of gradual worsening of disability outside of relapses [2]. Evidence suggests that MS is more prevalent in women than in men [4]. Most MS patients experience clinical disease onset between 20 and 40 years of age [4]. Several epidemiological studies have reported an increasing MS prevalence with increasing latitude. North European countries and North America constitute the high-risk MS prevalence zone, with a high MS prevalence of more than 100 cases per 100,000 population. Low MS risk areas are centred around the equator, with less than 30 cases per 100,000 population. Medium MS risk areas are located in between with prevalence within a similar range [5].

Observational studies have consistently demonstrated a higher clinical and economic burden owing to SPMS among all subtypes of MS [6, 7]. However, epidemiological data for SPMS are not available, and there is a great need to better understand the approximate prevalence of SPMS to estimate the true SPMS disease burden. In a consensus paper, Lublin et al. revised the definitions of the clinical course of MS by using refined descriptors that include consideration of disease activity and encourage differentiation between the relapsing and progressive forms of MS, but they also acknowledged that to date, there are no clear clinical, imaging, immunologic, or pathologic criteria to determine the transition point when RRMS converts to SPMS and that the transition is usually gradual [2]. With more clarity on the MS disease classification, researchers are currently attempting to explore epidemiological aspects by MS subtype [2, 8]. Khurana et al. reported a wide variation in the estimated prevalence of SPMS within and across countries but with uncertainty related to methodology and consequent results [9]. The objective of the current study was to estimate the prevalence of SPMS in the United States of America (USA), Europe, Canada, Australia, and Brazil based on the data collected from a systematic literature review. These countries were selected based on the availability and quality of MS prevalence data [10].

## Methods

### Data sources and search strategy

A systematic literature search of the Medline and Embase databases was performed using the OVID™ SP platform. Major European conference abstracts between 2016 and 2018 were also searched. The search strings used were “(Multiple sclerosis AND (Epidem* OR Inciden* OR Prevalen*).ti,ab. AND (Europe OR Europ* OR Albania OR Andorra OR Armenia OR Austria OR Azerbaijan OR Belarus OR Belgium OR Bosnia OR Herzegovina OR Bulgaria OR Croatia OR Cyprus OR Czech Republic OR Denmark OR Estonia OR Finland OR France OR Georgia OR Germany OR Greece OR Hungary OR Iceland OR Ireland OR Northern Ireland OR Eire OR Italy OR Kazakhstan OR Kosovo OR Latvia OR Liechtenstein OR Lithuania OR Luxembourg OR Macedonia OR Malta OR Moldova OR Monaco OR Montenegro OR Netherlands OR Norway OR Poland OR Portugal OR Romania OR Russia OR San Marino OR Serbia OR Slovakia OR Slovenia OR Spain OR Sweden OR Switzerland OR Turkey OR Ukraine OR United kingdom OR UK OR England OR Scotland OR Wales OR US OR United states OR Canada OR Australia OR Brazil)).mp.” To validate the search further, bibliographies of all relevant reviews and primary studies were screened.

### Inclusion and exclusion criteria

Studies published in English from database inception up to September 22, 2020, reporting the prevalence and/or incidence of adult MS (aged > 18 years) and the proportion of SPMS patients were included. Studies presenting paediatric MS data or MS epidemiological studies that did not include the proportion of SPMS patients were excluded. The study design was not a criterion for exclusion.

### Screening strategy and data extraction

After removing duplicates across the databases, the search result from the OVID platform was exported into an automated Excel file for screening. Two reviewers (VV and VK) independently screened the titles and abstracts and selected potentially relevant studies. Further, full texts of these studies were screened for inclusion and exclusion criteria. Reasons for exclusion were recorded, and any disparities in relevance were resolved by a third reviewer. Study details including region, target population, study design, diagnostic criteria, sampling method, date of survey, and study duration; baseline characteristics of the study population; and study outcomes (incident cases, incidence, prevalent cases, prevalence, and denominator used) were extracted into a predefined Excel data sheet.

### Quality assessment

The Loney quality assessment checklist, developed specifically for prevalence studies, was used for the quality grading of the included studies [11]. The Loney tool evaluates the methods of sampling, sample size, outcome measurement, outcome assessment, response rate, statistical reporting, and interpretation of study results. The overall single quality scores range from 0 to 8, with scores from 0 to 3 indicating poor, scores from 4 to 5 indicating moderate, and scores from 6 to 8 indicating higher methodological quality.

### Data analysis

Only moderate- and high-quality studies (i.e., scores from 4 to 8) were included in the meta-analysis. The meta-analysis was conducted using meta-analysis of proportions using “meta,” “metafor,” and “weightr” packages in the R software (version 3.5.2) [12, 13]. A random effects model was considered more appropriate for the present analysis owing to the heterogeneous study populations from diverse geographies. A binary outcome was assigned to each study based on the number of prevalent SPMS cases across the entire population. A pooled effect size estimate was evaluated for the studies by considering a weighted average of effect sizes, wherein weights were assigned proportionally to the sample size of each study. The Q, Ƭ^2^, and I^2^ statistics were measured to assess heterogeneity among the studies. The Q statistic is calculated as the weighted sum of squared differences between individual study effects and the pooled effect across studies. The Ƭ^2^ statistic is an estimate of between-study variance, whereas the I^2^ statistic is expressed as the percentage of the total variability in a set of effect sizes owing to true heterogeneity. If the Q, Ƭ^2^, and I^2^ values fell outside their 95% confidence interval (CI), 99% CI was used instead. The raw prevalence rates were transformed using the Freeman-Tukey (double arcsine) transformation to normalise their sampling distribution and stabilise their variance. A back transformation on the effect size was implemented using the same method to obtain the prevalence of SPMS.

Further, studies considered as outliers and influential on the summary effect size were identified by conducting tests such as studentised residuals test and leave-one-out analysis, presented in the Baujat plot [14]. Additionally, a diagnostic test was conducted to identify the influential studies. If substantial heterogeneity remained after excluding the outliers, a moderator analysis or subgroup analysis was conducted to discover other possible sources of heterogeneity. As meta-analysis of proportions includes observational and noncomparative studies, publication bias is not pertinent. However, the funnel plot and Egger test [15] were conducted to examine if the distribution of effect size estimates followed the usual pattern of less variation with higher number of studies and if the small-study effect was present.

### Ethical statement

The study did not require informed consent or institutional review board approval as no identifiable patient information was extracted. This systematic review was conducted and reported according to the Meta-analysis Of Observational Studies in Epidemiology (MOOSE) guidelines and the Preferred Reporting Items for Systematic Reviews and Meta-Analysis (PRISMA) statement [16, 17]. The review protocol is available with the corresponding author.

## Results

**Fig. 1:**
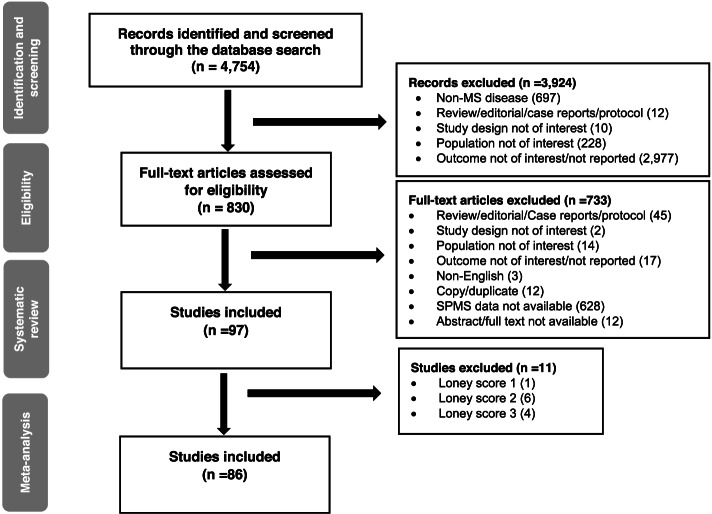
Flow diagram of the article selection process. MS, multiple sclerosis; SPMS, secondary progressive multiple sclerosis

A total of 4754 articles were retrieved from the search, of which 97 relevant studies were included and reviewed for their quality using the Loney score. Following quality assessment, 86 moderate- and high-quality studies were included in the meta-analysis (Fig. 1). Most included studies were retrospective chart reviews that followed the Poser or McDonald criteria for diagnosis (75 studies). Most studies were conducted in European countries (84 studies), especially Italy (19 studies) and Spain (13 studies). None of the epidemiological studies from the USA reported the proportion of SPMS patients; hence, they were not included in this review (Table 1). The average Loney score for all the 97 included studies was 4.6 and ranged from 1 to 8. A total of 86 studies scored ≥4 on the Loney scale and were included in the meta-analysis (Additional file 1, Table 1). Only one study reported the proportion of SPMS patients according to its subtypes. The estimated prevalence of SPMS with progression but without activity was 3.4/100,000 and without progression or activity was 6.9/100,000 [18].

**Table 1:** Summary of SPMS prevalence studies and quality assessments

	Studies	Region	Study year/ prevalence day	Loney score	Diagnostic criteria	General population denominator	SPMS prevalent cases	SPMS prevalence (per 100,000)	MS prevalence (per 100,000)
**Europe**									
**Bosnia and Herzegovina**									
1	Klupka-Saric et al., 2007 [19]	Western Herzegovina	Dec 31, 2003	5	McDonald criteria	300,746	29	9.6	26.9
2	Klupka-Saric and Galic, 2010 [20]	Western Herzegovina Canton and Herzegovina-Neretva Canton	Dec 31, 2006	5	McDonald criteria	309,712	29	9.4	31
**Bulgaria**									
3	Milanov et al., 1999 [21]	Sofia	Mar 31, 1998	5	Poser criteria	74,334	12	16.1	43.1
		Samokov				44,616	10	22.4	38.1
**Croatia**									
4	Perkovic et al., 2010 [22]	The town of Cabar	Dec 31, 2001	3	NR	4387	3	59.5	205.7
**Croatia, Slovenia**									
5	Peterlin et al., 2006 [23]	Gorski Kotar, Croatia and Kocˇevje, Slovenia	Jun 1, 1999	5	Poser criteria	57,258	35	61.8	151.9
**Finland**									
6	Laakso et al., 2019 [24]	Helsinki and Uusimaa, Southwest Finland, Tavastia Proper, Northern Savonia, and Central Finland	Dec 31, 2018	5	McDonald criteria	2,804,616	723	25.8	191.3
**France**									
7	Berr et al., 1989 [25]	Hautes-Pyrenees	Jun 1, 1983	2	Poser criteria	227,942	5	2.2	40.0
8	Debouverie, 2009 [26]	Lorraine	2002	4	Diagnosed by a neurologist	2,310,376	933	40.4	109.0
**Germany**									
9	Fasbender and Kolmel, 2008 [27]	Urban Area of Erfurt, Thuringia	Jan 31, 2006	4	Poser criteria	201,267	97	48.3	127.2
10	Hoer et al., 2014 [28]	Bavaria	2009	2	Diagnosed by a neurologist	10,400,000	1363	13.1	174.8
**Greece**									
11	Papathanasopoulos et al., 2008 [29]	Rion-Patras	Dec 31, 2006	2	Poser and McDonald criteria	652,108	172	26.4	119.61
12	Piperidou et al., 2003 [30]	Province of Evros	Dec 31, 1999	1	Poser criteria	143,752	14	9.7	38.9
**Hungary**									
13	Bencsik et al., 1998 [31]	Szeged city, Csongra’d County	1997	4	Poser criteria	198,682	5	2.6	65.0
14	Bencsik et al., 2001 [32]	Csongrad County	Jul 1, 1999	4	Poser criteria	400,128	48	12.0	62.0
15	Zsiros et al., 2014 [33]	Csongrad County	Jan 1, 2013	4	McDonald criteria	421,827	52	12.3	89.8
16	Biernacki et al., 2020 [34]	Csongrad County	Jan 1, 2019	4	McDonald criteria	399,012	102	25.6	105.3
**Ireland**									
17	McDonnell and Hawkins, 1998 [35]	Ballymena, Coleraine, Ballymoney, and Moyle districts spanning the counties of Antrim and Derry in Northern Ireland	July 1, 1996	5	Poser criteria	151,000	111	73.5	190.0
18	McGuigan et al., 2004 [36]	Wexford	Jan 1, 2001	5	Poser criteria	104,372	49	46.6	120.7
		Donegal				129,994	92	70.5	184.6
19	Gray et al., 2008 [37]	Northeast of Northern Ireland	Jan 1, 2004	6	Poser/McDonald criteria	160,446	112	69.8	230.6
20	Lonergan et al., 2011 [38]	Donegal County	Dec 31, 2007	6	McDonald criteria	113,347	124	109.1	290.3
		Wexford				119,442	75	62.6	144.8
		Southeast Dublin city				101,721	51	50.1	127.8
		All three areas				334,510	251	75.0	188.9
**Italy**									
21	Bellantonio et al., 2013 [39]	Campobasso, chief town of Molise region	Sep 30, 2009	5	Diagnosed by a neurologist	51,633	17	32.9	91.0
22	Bergamaschi et al., 2020 [40]	Pavia, Northern Italy	Dec 31, 2016	5	McDonald criteria	547,251	295	53.9	169.4
23	Caniglia-Tenaglia et al., 2018 [41]	Republic of San Marino	Dec 31, 2014	5	Diagnosed by a neurologist	32,789	12	36.8	204.3
24	Cavalletti et al., 1994 [42]	Province of Modena, Northern Italy	Dec 31, 1990	5	McAlpine and Confavreux	603,989	53	8.8	38.9
25	Granieri et al., 1996 [43]	Ferrara	Dec 31, 1993	5	Poser criteria	358,808	73	20.3	69.4
26	Granieri et al., 2007 [44]	Province of Ferrara, Northern Italy	Dec 31, 2004	5	Poser criteria	349,777	147	41.9	120.9
27	Granieri et al., 2008 [45]	Republic of San Marino	Dec 31, 2005	4	Poser criteria	29,999	5	16.7	166.7
28	Granieri et al., 2018 [46]	Province of Ferrara, Northern Italy	Dec 31, 2016	4	McDonald 2010 criteria	351,436	162	45.9	197.5
29	Grimaldi et al., 2007 [47]	Caltanissetta (Sicily), Southern Italy	Dec 31, 2002	4	Poser criteria	60,919	16	26.3	165.8
30	Guidetti et al., 1995 [48]	Provinces of Reggio Emilia and Modena	Dec 31, 1990	4	McAlpine criteria	1,024,223	30	2.9	40.2
31	Iuliano et al., 2014 [49]	Salerno (Southern Italy)	Dec 31, 2010	5	McDonald criteria	366,025	79	21.6	85.2
32	Millefiorini et al., 2010 [50]	Province of Frosinone	Jan 1, 2007	5	Poser criteria	491,548	85	17.2	95.0
33	Nicoletti et al., 2001 [51]	Catania, Sicily	Jan 1, 1995	5	Poser criteria	333,075	68	20.4	58.5
34	Nicoletti et al., 2005 [52]	Catania, Sicily	Dec 31, 1999	5	Poser criteria	313,110	77	24.6	92.0
35	Nicoletti et al., 2011 [53]	Catania, Sicily	Dec 31, 2004	5	Poser criteria	313,110	90	28.7	127.1
36	Patti et al., 2019 [54]	Sicily	Dec 31, 2018	4	McDonald criteria and Thompson criteria	23,948	5	20.9	292.3
37	Solaro et al., 2005 [55]	Province of Genoa	Dec 31, 1997	5	Poser criteria	913,218	150	16.4	94.0
38	Totaro et al., 2000 [56]	L’Aquila	Dec 31, 1996	5	Poser criteria	297,828	29	9.7	53.0
**Kosovo**									
39	Zeqiraj et al., 2014 [57]	Prishtina	2003–2012	2	McDonald criteria	2,102,041	93	4.4	19.6
**Netherlands**									
40	Minderhoud et al., 1988 [58]	Groningen	NR	2	Poser criteria	560,000	108	19.3	61.1
**Norway**									
41	Dahl et al., 2004 [59]	Nord-Trøndelag County	Jan 1, 2000	6	Diagnosed by a neurologist	127,108	59	46.4	163.6
42	Gronning and Mellgren, 1985 [60]	Troms and Finnmark	Jan 1, 1983	3	Rose criteria	225,073	23	10.4	31.5
43	Risberg et al., 2011 [61]	Oppland County hospitals, Gjøvik, and Lillehammer	Jan 1, 2002	4	Poser criteria	183,235	95	51.8	174.1
**Poland**									
44	Brola et al., 2016 [62]	Swietokrzyskie Province	Dec 31, 2014	5	McDonald criteria	1,263,176	317	25.1	115.7
45	Brola et al., 2017 [63]	Swietokrzyskie Province	Dec 31, 2015	5	McDonald criteria	1,257,179	360	28.6	121.3
46	Kapica-Topczewska et al., 2018 [64]	Central Poland	Dec 31, 2013	5	McDonald criteria	1,268,239	311	24.6	109.1
		Northeastern Poland				1,195,625	318	26.6	108.6
47	Kułakowskaa et al., 2017 [65]	Northeastern Poland (Podlaskie voivodeship)	NR	3	McDonald criteria	750,460	200	26.6	108.6
48	Potemkowski and Jasinska, 2015 [66]	Kielce, Central Poland	NR	4	McDonald criteria	200,938	71	35.4	98.53
**Portugal**									
49	Branco et al., 2020 [67]	Entre Douro e Vouga region	Jul 1, 2014	5	McDonald criteria	274,859	36	13.1	64.4
50	De Sa et al., 2006 [68]	District of Santarém	Nov 1, 1998	6	Poser criteria	62,621	6	9.6	46.3
51	Figueiredo et al., 2015 [69]	Braga	Dec 31, 2009	5	Diagnosed by a neurologist	866,012	49	5.7	39.8
52	Lopes et al., 2020 [70]	Sao Miguel	Jul 1, 2019	4	McDonald criteria	137,150	4	2.9	34.3
53	Ruano et al., 2014 [71]	Entre Douro-e-Vouga	Jan 1, 2013	4	McDonald criteria	274,859	34	12.3	58.6
**Romania**									
54	Becus and Popoviciu, 1994 [72]	Mures County	Dec 31, 1986	2	Diagnosed by a neurologist	615,032	8	1.3	21.0
55	Cornea et al., 2016 [73]	Timis County	Aug 16, 2016	3	McDonald criteria	486,420	45	9.3	69.1
**Serbia**									
56	Pekmezovic et al., 2019 [74]	Belgrade	Dec 31, 2018	4	McDonald criteria	1,685,673	586	34.7	136.8
57	Toncev et al., 2011 [75]	Sumadija	Dec 31, 2006	4	McDonald criteria	298,778	62	20.8	64.9
**Spain**									
58	Aladro et al., 2005 [76]	Las Palmas, Canary Islands	Dec 31, 2002	5	Poser criteria/McDonald criteria	82,623	12	14.5	73.8
59	Benito-Leon et al., 1998 [77]	Mostoles	Feb 1, 1998	5	Poser criteria	195,979	9	4.6	43.4
60	Bufill et al., 1995 [78]	Region of Osona in northern Catalonia	Dec 31, 1991	4	Poser criteria	71,985	6	8.3	58.0
61	Candeliere-Merlicco et al., 2016 [79]	Health District III, Murcia	Dec 13, 1999	5	McDonald criteria	171,040	27	15.8	71.9
62	Casquero et al., 2001 [80]	Menorca (Balearic Islands)	Dec 31, 1996	5	Poser criteria	67,009	9	13.4	68.6
63	Costa Arpin et al., 2020 [81]	Santiago de Compostela	Dec 31, 2015	5	McDonald criteria	95,612	24	25.1	152
64	Hernandez, 2002 [82]	Island of La Palma, Canary Islands	Dec 15, 1998	4	Poser criteria	81,507	11	13.5	41.7
65	Izquierdo et al., 2015 [83]	Northern Seville	Dec 31, 2011	5	Poser criteria	163,324	24	14.7	90.2
66	Modrego Pardo et al., 1997 [84]	Province of Teruel	Mar 1, 1996	5	Poser criteria	143,680	4	2.8	32.0
67	Modrego and Pina, 2003 [85]	Bajo Aragon, province of Teruel, Northeastern Spain	Jan 1, 2003	5	Poser criteria	58,666	5	8.5	75.0
68	Perez-Carmona et al., 2017 [18]^a^	San Vicente del Raspeig	Apr 10, 2017	3	2010 McDonald criteria- MS diagnosis, Lublin criteria (2013 revisions) – MS subtypes	56,696	NR	SPMS with progression but without activity: 3.4%; SPMS without progression or activity: 6.9%	102.3
69	Perez-Carmona et al., 2019 [86]	San Vicente del Raspeig	Dec 31, 2018	5	McDonald criteria	1,685,673	586	34.7	136.8
70	Pina et al., 1998 [87]	Sanitary District of Calatayud	Apr 1, 1995	5	NR	58,591	18	30.7	58.0
71	Tola et al., 1999 [88]	Valladolid	Mar 1, 1997	5	Poser criteria	92,632	6	6.5	58.3
**Sweden**									
72	Bostrom et al., 2009 [89]	County of Varmland in Western Sweden	Dec 31, 2002	4	Poser criteria	273,419	205	75.0	170.1
**Turkey**									
73	Akdemir et al., 2017 [90]	Middle Black Sea Region	Aug 2010–May 2011	4	McDonald criteria	3,666,667	74	2.0	43.2
74	Çelik et al., 2011 [91]	Edirne City	2003	5	McDonald criteria	119,298	8	6.7	33.9
	Çelik et al., 2011 [91]		2004			119,298	8	6.7	36.5
75	Gokce et al., 2019 [92]	Sivas Province	Apr 2017–Jan 2018	7	McDonald criteria	6595	4	60.7	288
76	Turk Boru et al., 2006 [93]	Maltepe, Istanbul	Nov 2002–May 2003	7	Poser criteria	32,531	11	33.8	101.4
77	Turk Boru et al., 2011 [94]	Three areas of the Black Sea coast of Turkey (Kandıra, Geyve, Erbaa)	2006 and 2010	8	Poser criteria	53,364	9	16.9	50.6
78	Turk Boru et al., 2018 [95]	Gazipasa (Mediterranean coast)	Apr–May 2012	7	McDonald 2010 criteria	13,451	1	7.4	52.0
		Artvin (Black sea coast)	May–Jun 2012			16,116	1	6.2	18.6
		Ordu (Black sea coast)	Nov–Dec 2012			28,800	4	13.9	55.5
79	Turk Boru et al., 2020 [96]	Eregli	May–Oct 2018	7	McDonald criteria	32,261	5	15.5	96.1
	Turk Boru et al., 2020 [96]	Devrek				21,963	2	9.1	45.5
**United Kingdom**									
80	Ford et al., 1998 [97]	Leeds	Apr 30, 1996	4	Poser criteria	732,061	225	30.7	97.3
81	Ford et al., 2002 [98]	Leeds	Oct 31, 1999	5	Poser criteria	732,061	244	33.3	108.7
82	Fox et al., 2004 [99]	Devon	Jun 1, 2001	5	Poser criteria	341,796	121	35.3	117.6
83	Gajofatto et al., 2013 [100]	Verona	Dec 31, 2001	5	McDonald criteria	253,208	59	23.4	106.0
84	Robertson et al., 1995 [101]	Cambridgeshire	Jul 1, 1993	5	Poser, Allison, and Millar criteria	378,959	85	22.4	118.0
85	Simpson et al., 2015 [102]	Isle of Man	2006	5	McDonald criteria	80,058	56	69.9	153.6
			2011			84,497	63	74.6	179.9
86	Visser et al., 2012 [103]	Aberdeen, Orkney, Shetland	Sep 24, 2009	5	Poser and McDonald criteria	24,8102	237	95.5	238.0
**Australia**									
87	Barnett et al., 2003 [104]	Newcastle	1996	5	Diagnosed by a neurologist	133,686	13	9.7	59.1
88	Ribbons et al., 2017 [105]	Newcastle	Aug 9, 2011	5	McDonald criteria	148,535	17	11.8	124.2
**Brazil**									
89	Callegaro et al., 2001 [106]	Sao Paulo	Jul 1, 1997	4	Poser criteria	9,380,000	23	0.2	15.8
90	Calmon et al., 2016 [107]	Volta Redonda	Nov 2012	5	Poser criteria/McDonald criteria	260,180	1	0.4	15.4
91	Negreiros et al., 2015 [108]	João Pessoa, Paraíba	Jul 2013	4	Diagnosed by a neurologist	723,515	19	2.6	12.0
92	Lana-Peixoto et al., 2012 [109]	Belo Horizonte	July 1, 2001	4	Poser criteria	2,238,526	78	3.5	18.1
93	Ribeiro et al., 2011 [110]	Uberaba, Minas Gerais	Aug–Dec 2008	4	Poser and McDonald criteria	287,760	2	0.7	12.5
94	Ribeiro et al., 2019 [111]	Goiânia	Dec 31, 2015	4	Poser or McDonald criteria	1,430,697	27	1.9	22.2
**Canada**									
95	Sloka et al., 2005 [112]	Newfoundland and Labrador	Dec 31, 2001	5	Poser criteria	521,986	94	18.0	94.4
96	Warren and Warren, 1992 [113]	County of Barrhead, Alberta	Jan 1, 1990	5	Poser criteria	9720	9	92.6	196.0
97	Warren and Warren, 1993 [114]	Westlock county	Jan 1, 1991	5	Poser criteria	11,510	9	78.2	200.0

^a^As this is the sub-group analysis, not included in the meta-analysis

*MS* Multiple sclerosis, *NR* Not Reported, *SPMS* Secondary progressive multiple sclerosis

### Australia

**Fig. 2:**
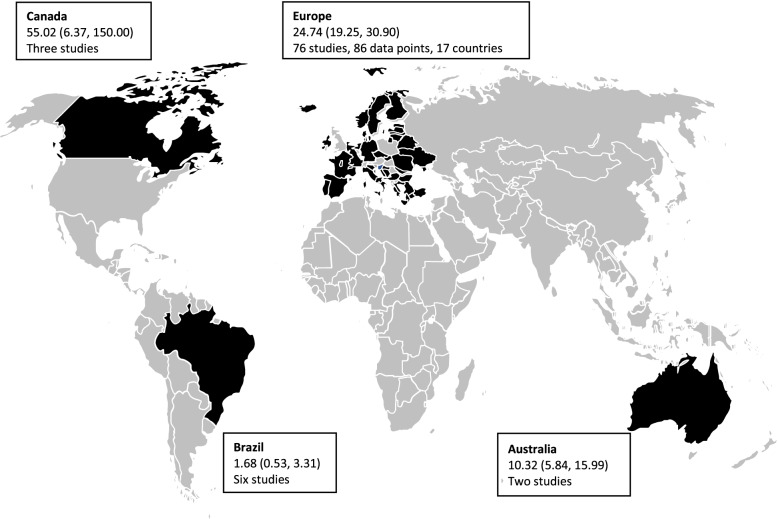
Estimated pooled prevalence (per 100,000 [99% CI]) of SPMS across countries. CI, confidence interval; SPMS, secondary progressive multiple sclerosis

Two moderate-quality studies from Newcastle were included in this meta-analysis [104, 105]. The pooled prevalence of SPMS in Australia was 10.32 (99% CI: 5.84, 15.99)/100,000 (Fig. 2). The MS prevalence has increased by 110% between 1996 and 2011 in Newcastle. However, the SPMS prevalence has increased by only 22%. Different diagnostic criteria were used in these studies [104, 105] (Additional file 1, Fig. 1).

### Brazil

Six moderate-quality studies were included in this meta-analysis [106–111]. All studies reported a very low MS prevalence and proportion of SPMS patients. The pooled prevalence of SPMS was 1.68 (99% CI: 0.53, 3.31)/100,000 [106–111] (Table 1 and Fig. 2).

### Canada

Three moderate-quality studies were included in this meta-analysis [112–114]. Only the Poser diagnostic criteria were used in all the studies. Two studies conducted in the early 90s in the counties of Westlock and Barrhead reported a very high MS and SPMS prevalence [113, 114]. Another study published in 2005 reported a low SPMS prevalence in the region of Newfoundland and Labrador [112]. The pooled prevalence of SPMS was 55.02 (99% CI: 6.37, 150.00)/100,000 (Table 1 and Fig. 2).

### Europe

**Fig. 3:**
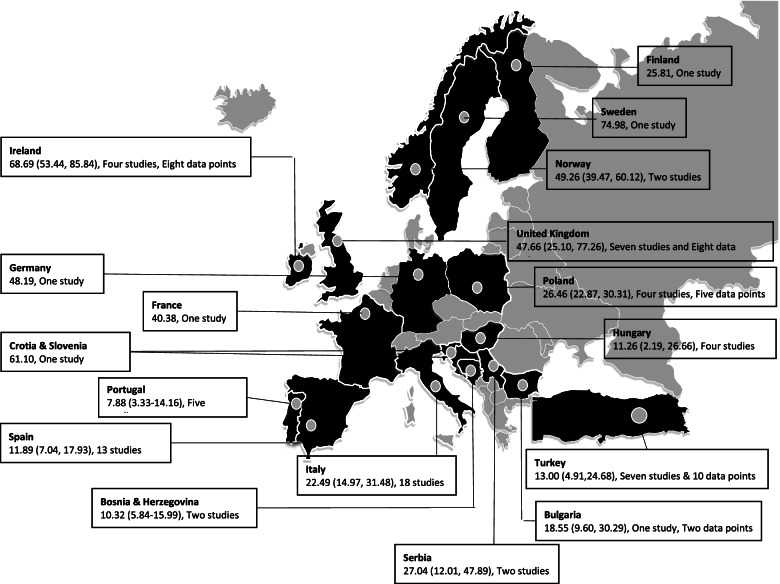
Estimated pooled prevalence (per 100,000 [99% CI]) of SPMS across European countries. CI, confidence interval; SPMS, secondary progressive multiple sclerosis

The pooled SPMS prevalence in European countries was 24.74 (99% CI: 19.25, 30.90)/100,000 (Fig. 2). Among the European countries, the estimated pooled prevalence of SPMS was highest in Sweden and lowest in Portugal. North European countries such as Sweden, Norway, United Kingdom (UK), and Ireland reported a higher SPMS prevalence than the rest of the European countries. The only exception was a study conducted in Croatia and Slovenia, which reported a higher prevalence equivalent to that in the North European countries in these two countries despite being South European countries (Fig. 3). Low-quality studies from Greece, Kosovo, Netherlands, and Romania were not included in this meta-analysis.

#### Bosnia and Herzegovina

Two moderate-quality studies were included in this meta-analysis [19, 20]. Both studies used the McDonald diagnostic criteria. The pooled prevalence of SPMS was 10.32 (99% CI: 5.84, 15.99)/100,000 (Fig. 3). Between 2003 and 2006, the MS prevalence increased by 15%, while the SPMS prevalence decreased by 2% (Table 1) [19, 20].

#### Bulgaria

Only one moderate-quality study was included in this meta-analysis [21]. Even though MS patients were more prevalent in the Sofia region than in the Samokov region, the prevalence of SPMS was higher in the Samokov region compared with the Sofia region (Table 1) [21]. The pooled prevalence of SPMS was 18.55 (99% CI: 9.60, 30.29)/100,000 (Fig. 3).

#### Croatia and Slovenia

Two MS epidemiological studies reported the proportion of SPMS patients [22, 23]. The study conducted by Perkovic et al. in Croatia did not meet the quality standards required for inclusion in this meta-analysis [22], while the study conducted by Peterlin et al. in Croatia and Slovenia was of moderate quality and was included in this meta-analysis [23]. This study reported a very high MS prevalence and a proportion of SPMS patients almost similar to that in the North European countries (Table 1 and Fig. 3).

#### Finland

One moderate-quality study conducted in 2018 was included in this meta-analysis [24]. The estimated SPMS prevalence was 25.81/100,000 (Table 1 and Fig. 3) [24].

#### France

One moderate-quality study was included in this meta-analysis [26]. This study reported an SPMS prevalence of 40.38/100,000 [26]. Another study was excluded from the meta-analysis owing to low quality [25]. In France, over a period of 19 years, the SPMS prevalence has increased by 18.4 times, while the MS prevalence has increased only by 2.7 times (Table 1 and Fig. 3).

#### Germany

Two MS epidemiological studies reported the proportion of SPMS patients [27, 28], one among them was of moderate quality and was included in this meta-analysis [27]. In 2006, the SPMS prevalence was 48.19/100,000 and MS prevalence was 127.2/100,000 in the urban area of Erfurt (Table 1 and Fig. 3).

#### Hungary

Four moderate-quality studies conducted in Csongrad County were included in this meta-analysis [31–34]. The pooled prevalence of SPMS was 11.26 (99% CI: 2.19, 26.66)/100,000 (Fig. 3). The prevalence of SPMS was 4.6 times greater in Csongrad County compared with the Szeged region of Hungary [31, 32]. Over a period of 14 years, the SPMS prevalence has remained almost same in Csongrad County, while the MS prevalence has increased by 45% [32, 33]. A recent study conducted in Csongrad County in early 2019 showed a two times increase in the SPMS prevalence and a 1.2 times increase in the MS prevalence since 2013 [34] (Additional file 1, Fig. 2).

#### Ireland

A total of four moderate- and high-quality studies were included in this meta-analysis [35–38]. The pooled prevalence of SPMS was 68.69 (99% CI: 53.44, 85.84)/100,000 (Fig. 3). The prevalence of SPMS was highest in Donegal County in the year 2007 and lowest in Wexford County in the year 2001 (Table 1). Over a period of 6 years, the SPMS prevalence has increased by 34% and 55% in the Wexford and Donegal counties, respectively. The increase in SPMS prevalence was in line with that of overall MS prevalence in Donegal County but not in Wexford County (Additional file 1, Fig. 3) [36, 38].

#### Italy

**Fig. 4:**
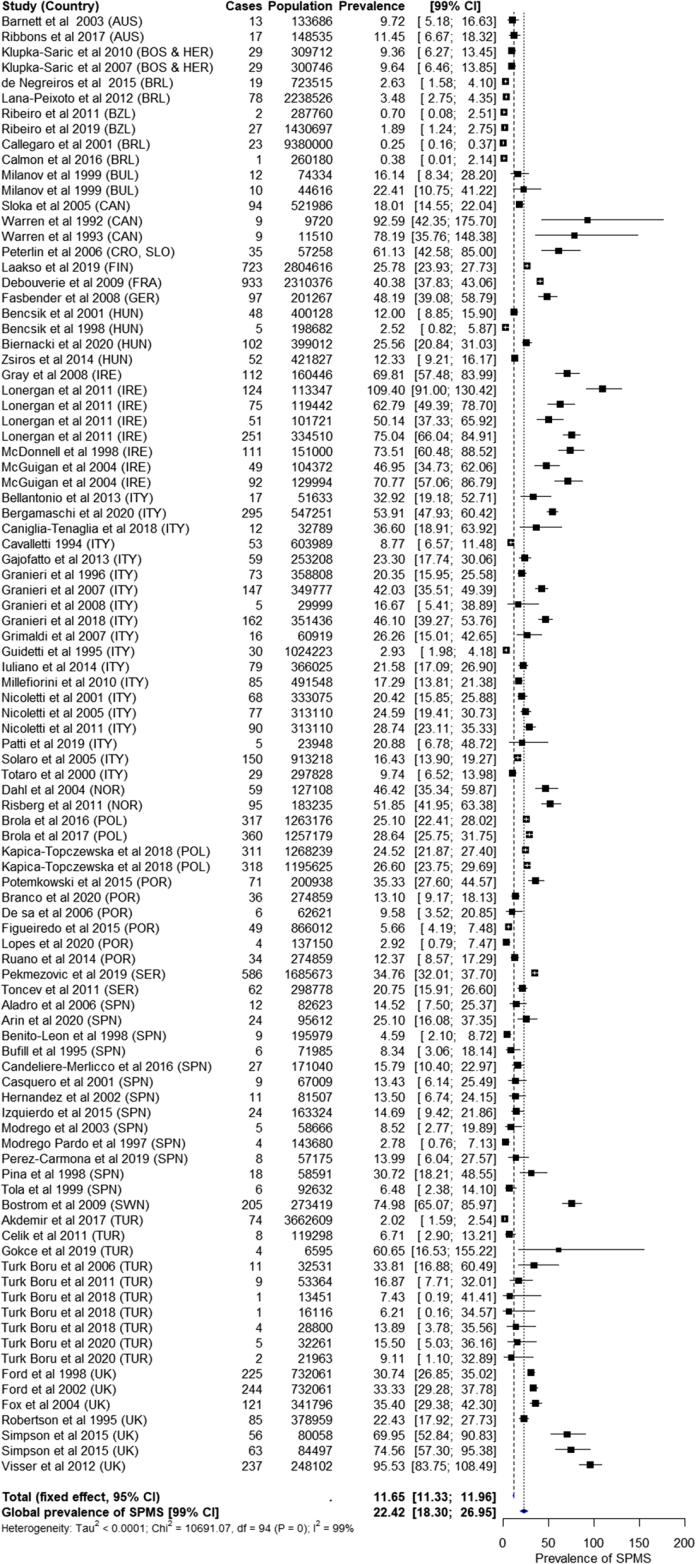
Estimated pooled prevalence (per 100,000 [99% CI]) of SPMS across countries. Country codes: Australia (AUS), Bosnia and Herzegovina (BOS & HER), Brazil (BZL), Bulgaria (BUL), Canada (CAN), Croatia, Slovenia (CRO, SLO), Finland (FIN), France (FRA), Germany (GER), Hungary (HUN), Ireland (IRE), Italy (ITY), Norway (NOR), Poland (POL), Portugal (POR), Serbia (SER), Spain (SPN), Sweden (SWN), Turkey (TUR), United Kingdom (UK). CI, confidence interval; SPMS, secondary progressive multiple sclerosis

A total of 18 MS moderate-quality studies reported the proportion of SPMS patients and thus were included in this meta-analysis [39–56]. The pooled prevalence of SPMS was 22.49 (99% CI: 14.97, 31.48)/100,000 (Fig. 3). Multiple studies conducted in the province of Ferrara, Republic of San Marino, and Catania showed an increase in the SPMS prevalence over time. Between 2001 and 2011, a gradual increase in the SPMS prevalence was observed in Catania, while the increase in MS prevalence was more pronounced [51–53]. In the Republic of San Marino, between 2005 and 2014, the SPMS prevalence increased by 120%, while the MS prevalence increased by 22.5% [41, 45]. In the province of Ferrara, between 1993 and 2004, the SPMS prevalence increased by 106%, while the MS prevalence increased by 74% [43, 44]. In the same region, between 2004 and 2016, the SPMS prevalence increased only by 9.5%, while the MS prevalence increased by 63% [44, 46] (Table 1 and Additional file 1-Fig. 4).

#### Norway

Two of three studies were of moderate quality and were included in this meta-analysis [59, 61]. The pooled prevalence of SPMS was 49.26 (99% CI: 39.47, 60.12)/100,000 (Fig. 3).

#### Poland

Four of five studies were of moderate quality and were included in this meta-analysis [62–64, 66]. The pooled prevalence of SPMS was 26.46 (99% CI: 22.87, 30.31)/100,000 (Fig. 3). During the 1-year period in the Swietokrzyskie Province, the MS prevalence increased by 5% and SPMS prevalence increased by 14% (Additional file 1-Fig. 5) [62, 63].

#### Portugal

Five moderate-quality studies were included in this meta-analysis [67–71]. The pooled prevalence of SPMS was 7.88 (99% CI: 3.33, 14.16)/100,000. The SPMS prevalence increased with increase in MS prevalence (Fig. 3).

#### Serbia

Two moderate-quality studies were included in this meta-analysis [74, 75]. The pooled prevalence of SPMS was 27.04 (99% CI: 12.01, 47.89)/100,000 (Fig. 3 and Table 1).

#### Spain

A total of 14 MS epidemiological studies reported the proportion of SPMS patients [18, 76–88]. Of these, 13 were of moderate and high quality and thus were included in this meta-analysis [76–88]. The pooled prevalence of SPMS was 11.89 (99% CI: 7.04, 17.93)/100,000. The prevalence of SPMS varied between 2.8 and 34.7 cases per 100,000 and was the highest in San Vicente del Raspeig (Table 1 and Fig. 3).

#### Sweden

One moderate-quality study was included in this meta-analysis [89]. The estimated SPMS prevalence was 75.0/100,000, which was the highest among the included studies (Table 1 and Fig. 3).

#### Turkey

All seven studies were of moderate and high quality and were included in this meta-analysis [90–96]. The pooled prevalence of SPMS was 13.00 (99% CI: 4.91, 24.68)/100,000 (Table 1 and Fig. 3).

#### United Kingdom

A total of seven moderate-quality studies were included in this meta-analysis [97–103]. The pooled prevalence of SPMS was 47.66 (99% CI: 25.10, 77.26)/100,000 (Fig. 3). In Leeds, between 1996 and 1999, the MS prevalence increased by 12%, while the SPMS prevalence increased by 8.5% [97, 98]. In the Isle of Man, between 2006 and 2011, the SPMS prevalence increased by 7%, while the MS prevalence increased by 17% [102] (Additional file 1-Fig. 6).

### Worldwide

The overall pooled prevalence of SPMS was 22.42 (99% CI: 18.30, 26.95)/100,000 with substantial heterogeneity (Fig. 4). Publication bias assessed by constructing a funnel plot showed heterogeneity or small-study effect; however, the effect was not significant (*p* = 0.334) (Additional file 1-Fig. 7). Brazil reported the lowest pooled prevalence, followed by Australia, Europe, and Canada (Fig. 2). Overall, the prevalence of SPMS correlated with that of MS (Pearson’s correlation coefficient: 0.89).

The SPMS prevalence varied widely among different regions within each country. In Hungary, between 1997 and 1999, the prevalence of SPMS increased by 4.6 times in the entire Csongrad County compared with that in the Szeged region of Csongrad County [31, 32]. Multiple studies conducted in the same regions over time have shown an increase in the prevalence of SPMS. The only exception was the study conducted in Bosnia and Herzegovina, which showed a slight reduction of 2% in the SPMS prevalence between 2003 and 2006 [19, 20]. The extent of increase in the SPMS prevalence varied based on the diagnostic criteria used. Studies using the same diagnostic criteria reported a moderate increase in the SPMS prevalence ranging between 7% and 20.5% [51–53, 62, 63, 97, 98, 102]. The only exceptions were two Italian studies conducted in the province of Ferrara between 1993 and 2004 that used the Poser diagnostic criteria, which showed a very high increase of 106% in the prevalence of SPMS [43, 44].

The overall prevalence of SPMS statistically correlated with the prevalence of MS. However, this correlation hypothesis was not consistent when focusing on the extent of correlation. Only in Donegal County, Ireland, the SPMS prevalence increased proportionately with that of MS [36, 38]. The proportion of increase in the SPMS prevalence was lower than that of MS prevalence in Newcastle, Australia [104, 105]; Csongrad County, Hungary [32, 33]; Catania, Italy [51–53]; Ferrara, Italy [44, 46]; Swietokrzyskie Province, Poland [62, 63]; and Isle of Man, UK [102]. The proportion of increase in the SPMS prevalence was higher than that of MS prevalence in the Republic of San Marino, Italy [41, 45]; Ferrara, Italy [43, 44]; and Leeds, UK [97, 98].

Access to oral disease-modifying therapies (DMTs) may have contributed to a decline in the SPMS prevalence. The estimated SPMS pooled prevalence in studies conducted before access to DMTs was 24.54 (CI: 17.50, 32.74)/100,000 in studies conducted between 1996 and 2010. The SPMS pooled prevalence in studies conducted after access to oral DMTs since 2011 was 18.24/100,000 (CI: 11.27, 26.82). Most studies used the Poser or McDonald diagnostic criteria (75 studies). The pooled SPMS prevalence in studies that used the Poser (22.55 [99% CI: 14.88, 31.76]/100,000) and McDonald (24.96 [99% CI: 16.38, 35.28]/100,000) diagnostic criteria was comparable.

Using various statistical tests mentioned earlier, a Brazilian study by Callegaro et al. 2001 [106], an Irish study by Lonergan et al. 2011 [38], and a UK study by Visser et al. 2012 [103] were identified as the most influential studies (Additional file 1-Table 2, Additional file 1-Figs. 8–9). The prevalence of SPMS after removing these three influential studies was 21.17 (99% CI: 17.90, 25.90)/100,000 compared with the previous result of 22.42 (99% CI: 18.30, 26.95)/100,000.

The subgroup analysis showed that the moderators such as world region (European vs. non-European countries) (Additional file 1-Fig. 10), introduction of oral DMTs (before 2010 vs. after 2010) (Additional file 1-Fig. 11), and sample size (≤100 vs. ≥100 and ≥ 1000) (Additional file 1-Fig. 12) were significantly (all *p* < 0.000001) associated with the overall pooled prevalence of SPMS. World region contributed to 10.95%, introduction of oral DMTs contributed to 0.81%, and sample size contributed to 22.13% of the total between-study variance. The moderator diagnostic criteria (McDonald or Poser criteria vs. others) (Additional file 1-Fig. 13) did not significantly influence the overall pooled prevalence of SPMS (*p* = 0.278) and contributed to only 0.21% of the total between-study variance.

## Discussion

Several MS epidemiological studies have been published across geographies. However, the same research interest has not been observed for the MS subtypes. A total of 92 countries accounting for 79% of the world population provided MS data for the Atlas of MS 2013 updates. On the contrary, studies from only 20 countries accounting for less than 10% of the world population contributed to the current SPMS prevalence systematic review [5]. This systematic literature review is an attempt to understand the epidemiology of SPMS in Australia, Brazil, Canada, European countries, and the USA. Our study was designed to reduce the uncertainty of outputs using a robust systematic methodology and the Loney quality grading of publications.

Most studies included in this review were of moderate quality, with publication bias per the Loney et al. checklist. However, statistically, no publication bias was observed. It is interesting to note that none of the MS epidemiological studies reported the prevalence of SPMS despite the large number of studies published. Hence, we have estimated the prevalence of SPMS based on the proportion of SPMS patients reported in the MS epidemiological studies. None of the MS epidemiological studies conducted in the USA reported the proportion of SPMS patients. Most studies were conducted in European countries, especially Italy and Spain.

In line with the prevalence of MS reported in the previous studies, the estimated SPMS prevalence varied widely across geographies and was the highest in Sweden (75/100,000) and lowest in Brazil (1.35/100,000) [5, 38, 106, 115, 116]. These results are similar to the findings of MS Atlas 2013, which reported that the highest prevalence of MS in Europe was in Sweden (189/100,000) [5]. Factors considered as possible modifiers of prevalence are differences in actual prevalence by population demographics, in latitude or longitude, in healthcare resourcing such as number of neurologists per 100,000 population, in definitions of SPMS or reimbursement, and in audit of DMTs across countries leading to different levels of diagnostic moral hazard for SPMS.

Our systematic review did not find any demographical data on SPMS, possibly due to lack of focus on the SPMS population in MS research. However, population density had no influence on the SPMS prevalence pattern across countries [117]. Only one study reported the proportion of SPMS patients without disease progression two times that of SPMS patients with disease progression [18]. However, these data need further investigation.

Geographical region, such as European countries and non-European countries, significantly (*p* < 0.000001) influenced the overall pooled prevalence of SPMS. One of the reasons for this influence was latitude; epidemiological studies have established variations in MS prevalence with latitude, and similar patterns were also observed in SPMS populations across continents [5]. The analysis from this review found that Brazil reported a seven times lower pooled prevalence of SPMS than Australia, a 19 times lower pooled prevalence of SPMS than Europe, and a 42 times lower pooled prevalence of SPMS than Canada. Within Europe, latitudinal influence was observed among northern countries like Sweden, Norway, UK, and Ireland and the remaining European countries. The only exceptions were Croatia and Slovenia, which reported a higher prevalence despite being South European countries. However, because only one study was conducted together in Croatia and Slovenia, this finding needs further investigation. Similarly, longitudinal influence on the prevalence among the West European countries was also observed. Portugal being the extreme West European country had the lowest SPMS prevalence among the European countries. The prevalence increased by 70.4% than that of Spain in France and by 126.3% than that of France in Germany. However, these observations are inconclusive, as they cannot be generalised across other European countries; some results directly conflict with any interpretation of the results based on latitude or longitude.

The overall SPMS prevalence has increased since the 1990s till the introduction of oral DMTs in the year 2010. This may be due to the possibility of the real SPMS prevalence being more than the reported prevalence, as no separate treatment interventions for SPMS patients were available until recently. The introduction of oral DMTs significantly influenced the overall pooled prevalence of SPMS (*p* < 0.000001). The prevalence of SPMS statistically correlated with that of MS. However, the extent of increase in the SPMS prevalence did not correlate with that of MS.

In the current review, the availability of medical resources, especially neurosurgeons and neurologists per 100,000 population, had no apparent effect on the differences in the SPMS prevalence across countries [118]. However, between different regions of some countries, medical resources may have a direct influence. In Germany, the prevalence of SPMS in the urban area of Erfurt in 2006 was 3.7 times higher than that in Bavaria in 2009 [27, 28]. In contrast, in the Republic of Ireland, high-income counties with better healthcare facilities such as Dublin and Wexford had a lower prevalence of SPMS compared with Donegal, which is a county with the lowest regional per capita [36, 38, 119].

MS research has evolved significantly since 2000 with the introduction of different diagnostic criteria and DMTs. However, these evolutions did not reflect in the prevalence pattern in this study. The use of well-accepted diagnostic criteria, such as the McDonald or Poser criteria, did not influence the overall pooled prevalence of SPMS statistically. Even the quality of the studies did not seem to have an impact on prevalence. Finally, a sample size of below 100 compared with above 100 and below 1000 also significantly influenced the overall pooled prevalence of SPMS (*p* < 0.000001).

Our literature search was limited to English-language publications; however, we manually screened the bibliography of the included publications and found no additional references from other languages. Hence, we believe that the possibility of missing prevalence data is low. Despite including higher-quality studies, the possibility of publication bias cannot be ruled out considering the variability in the quality of the studies included. In summary, this study provides information on the epidemiology of SPMS. To the best of our knowledge, no studies specifically report the epidemiology of SPMS. Our review found high variability in the estimated SPMS prevalence and the quality of the studies conducted with no obvious explanation for variability based on what is known of the SPMS disease physiology. Quality grading of SPMS prevalence studies does not appear to reduce the uncertainty associated with the results. These variations may therefore be due to the differences across healthcare systems in the reporting of SPMS and audit of treatments. It may be important to consider this context in the design of future epidemiological studies of SPMS. Focus on MS subtypes such as SPMS is warranted in high-quality MS epidemiological studies like the MS Atlas project and the Global Burden of Disease project for a better understanding of the prevalence of SPMS.

## Conclusions

The estimated prevalence of SPMS and the quality of the studies varied widely. Common confounding factors like latitude that are known to affect MS prevalence did not fully explain the wide range of inter-country and intra-country variability identified in the results.

## Supplementary Information

**Additional file 1: Table 1.** Loney quality assessment of SPMS prevalence studies. **Table 2.** Leave-one-out analysis result. **Figure 1.** SPMS prevalence (per 100,000) pattern in Australia. **Figure 2.** SPMS prevalence (per 100,000) pattern in Hungary. **Figure 3.** SPMS prevalence (per 100,000) pattern in Ireland. **Figure 4.** SPMS prevalence (per 100,000) pattern in Italy. **Figure 5.** SPMS prevalence (per 100,000) pattern in Poland. **Figure 6.** SPMS prevalence (per 100,000) pattern in the United Kingdom. **Figure 7.** Funnel plot. **Figure 8.** Baujat plot. **Figure 9.** Studentized residuals test. **Figure 10.** Scatter plot of moderator region. **Figure 11.** Scatter plot of moderator introduction of oral DMT. **Figure 12.** Scatter plot of moderator sample size. **Figure 13.** Scatter plot of moderator diagnostic criteria.

## Data Availability

All data generated or analysed during this study are included in this published article [and its supplementary information files].

## Abbreviations

CI: Confidence interval
DMT: Disease-modifying therapy
MS: Multiple sclerosis
RRMS: Relapsing-remitting multiple sclerosis
SPMS: Secondary progressive multiple sclerosis
UK: United Kingdom
USA: United States of America
