# Supplementary material for: Systematic literature review and meta-analysis of the prevalence of secondary progressive multiple sclerosis in the USA, Europe, Canada, Australia, and Brazil
Source: BMC Neurol. 2022 Aug 17;22:301. doi: 10.1186/s12883-022-02820-0 (PMC9382820; doi:10.1186/s12883-022-02820-0)

Supplementary Material

Table 1. Loney quality assessment of SPMS prevalence studies

| **Serial #** | **Studies** | **Assessment questions** | | | | | | | | **CA score** |
| --- | --- | --- | --- | --- | --- | --- | --- | --- | --- | --- |
|  |  | **Random sample or whole population** | **Unbiased sampling frame (i.e. census data)** | **Adequate sample size (>300 patients)** | **Measures were the standard 1 point** | **Outcomes measured by unbiased assessors** | **Adequate response rate (70%), refusers described** | **Confidence intervals, subgroup analysis** | **Study subjects described** |  |
| **Europe** | | | | | | | | | | |
| **Bosnia and Herzegovina** | | | | | | | | | | |
| 1 | Klupka-Saric et al., 2007 [30] | 0 | 0 | 1 | 1 | 1 | 0 | 1 | 1 | 5 |
| 2 | Klupka-Saric and Galic, 2010 [31] | 0 | 0 | 1 | 1 | 1 | 0 | 1 | 1 | 5 |
| **Bulgaria** | | | | | | | | | | |
| 3 | Milanov et al., 1999 [32] | 0 | 0 | 1 | 1 | 1 | 0 | 1 | 1 | 5 |
| **Croatia** | | | | | | | | | | |
| 4 | Perkovic et al., 2010 [33] | 0 | 0 | 1 | 1 | 1 | 0 | 0 | 0 | 3 |
| **Croatia, Slovenia** | | | | | | | | | | |
| 5 | Peterlin et al., 2006 [34] | 0 | 0 | 1 | 1 | 1 | 0 | 1 | 1 | 5 |
| **Finland** | | | | | | | | | | |
| 6 | Laakso et al., 2019 [35] | 0 | 1 | 1 | 1 | 1 | 0 | 0 | 1 | 5 |
| **France** | | | | | | | | | | |
| 7 | Berr et al., 1989 [36] | 0 | 0 | 1 | 0 | 0 | 0 | 0 | 1 | 2 |
| 8 | Debouverie, 2009 [37] | 0 | 0 | 1 | 1 | 1 | 0 | 0 | 1 | 4 |
| **Germany** | | | | | | | | | | |
| 9 | Fasbender and Kolmel, 2008 [38] | 0 | 0 | 1 | 1 | 1 | 0 | 0 | 1 | 4 |
| 10 | Hoer et al., 2014 [39] | 0 | 0 | 1 | 0 | 0 | 0 | 0 | 1 | 2 |
| **Greece** | | | | | | | | | | |
| 11 | Papathanasopoulos et al., 2008 [40] | 0 | 0 | 1 | 0 | 0 | 0 | 0 | 1 | 2 |
| 12 | Piperidou et al., 2003 [41] | 0 | 0 | 0 | 0 | 0 | 0 | 0 | 1 | 1 |
| **Hungary** | | | | | | | | | | |
| 13 | Bencsik et al., 1998 [42] | 0 | 0 | 1 | 1 | 1 | 0 | 0 | 1 | 4 |
| 14 | Bencsik et al., 2001 [43] | 0 | 0 | 1 | 1 | 1 | 0 | 0 | 1 | 4 |
| 15 | Zsiros et al., 2014 [44] | 1 | 0 | 1 | 1 | 0 | 0 | 0 | 1 | 4 |
| 16 | Biernacki et al., 2020 [45] | 0 | 0 | 1 | 1 | 1 | 0 | 0 | 1 | 4 |
| **Ireland** | | | | | | | | | | |
| 17 | McDonnell and Hawkins, 1998 [46] | 0 | 0 | 1 | 1 | 1 | 0 | 1 | 1 | 5 |
| 18 | McGuigan et al., 2004 [47] | 0 | 0 | 1 | 1 | 1 | 0 | 1 | 1 | 5 |
| 19 | Gray et al., 2008 [48] | 0 | 1 | 1 | 1 | 1 | 0 | 1 | 1 | 6 |
| 20 | Lonergan et al., 2011 [49] | 1 | 1 | 1 | 1 | 0 | 0 | 1 | 1 | 6 |
| **Italy** | | | | | | | | | | |
| 21 | Bellantonio et al., 2013 [50] | 0 | 0 | 1 | 1 | 1 | 0 | 1 | 1 | 5 |
| 22 | Caniglia-Tenaglia et al., 2018 [52] | 0 | 0 | 1 | 1 | 1 | 0 | 1 | 1 | 5 |
| 23 | Cavalletti et al., 1994 [53] | 0 | 0 | 1 | 1 | 1 | 0 | 1 | 1 | 5 |
| 24 | Granieri et al., 1996 [54] | 0 | 0 | 1 | 1 | 1 | 0 | 1 | 1 | 5 |
| 25 | Granieri et al., 2007 [55] | 0 | 0 | 1 | 1 | 1 | 0 | 1 | 1 | 5 |
| 26 | Granieri et al., 2008 [56] | 0 | 0 | 1 | 1 | 1 | 0 | 0 | 1 | 4 |
| 27 | Granieri et al., 2018 [57] | 0 | 0 | 1 | 1 | 1 | 0 | 0 | 1 | 4 |
| 28 | Grimaldi et al., 2007 [58] | 0 | 0 | 0 | 1 | 1 | 0 | 1 | 1 | 4 |
| 29 | Guidetti et al., 1995 [59] | 0 | 0 | 1 | 1 | 1 | 0 | 1 | 0 | 4 |
| 30 | Iuliano et al., 2014 [60] | 0 | 0 | 1 | 1 | 1 | 0 | 1 | 1 | 5 |
| 31 | Millefiorini et al., 2010 [61] | 0 | 0 | 1 | 1 | 1 | 0 | 1 | 1 | 5 |
| 32 | Nicoletti et al., 2001 [62] | 0 | 0 | 1 | 1 | 1 | 0 | 1 | 1 | 5 |
| 33 | Nicoletti et al., 2005 [63] | 0 | 0 | 1 | 1 | 1 | 0 | 1 | 1 | 5 |
| 34 | Nicoletti et al., 2011 [64] | 0 | 0 | 1 | 1 | 1 | 0 | 1 | 1 | 5 |
| 35 | Solaro et al., 2005 [66] | 0 | 0 | 1 | 1 | 1 | 0 | 1 | 1 | 5 |
| 36 | Totaro et al., 2000 [67] | 0 | 0 | 1 | 1 | 1 | 0 | 1 | 1 | 5 |
| 37 | Patti et al., 2019 [65] | 0 | 0 | 1 | 1 | 1 | 0 | 0 | 1 | 4 |
| 38 | Bergamaschi et al., 2020 [51] | 0 | 0 | 1 | 1 | 1 | 0 | 1 | 1 | 5 |
| **Kosovo** | | | | | | | | | | |
| 39 | Zeqiraj et al., 2014 [68] | 0 | 0 | 1 | 0 | 0 | 0 | 0 | 1 | 2 |
| **Netherlands** | | | | | | | | | | |
| 40 | Minderhoud et al., 1988 [69] | 0 | 0 | 1 | 0 | 0 | 0 | 0 | 1 | 2 |
| **Norway** | | | | | | | | | | |
| 41 | Dahl et al., 2004 [70] | 0 | 0 | 1 | 1 | 1 | 1 | 1 | 1 | 6 |
| 42 | Gronning and Mellgren, 1985 [71] | 0 | 0 | 0 | 1 | 1 | 0 | 0 | 1 | 3 |
| 43 | Risberg et al., 2011 [72] | 0 | 0 | 1 | 1 | 1 | 0 | 0 | 1 | 4 |
| **Poland** | | | | | | | | | | |
| 44 | Brola et al., 2016 [73] | 0 | 0 | 1 | 1 | 1 | 0 | 1 | 1 | 5 |
| 45 | Brola et al., 2017 [74] | 0 | 0 | 1 | 1 | 1 | 0 | 1 | 1 | 5 |
| 46 | Kapica-Topczewska et al., 2018 [75] | 0 | 0 | 1 | 1 | 1 | 0 | 1 | 1 | 5 |
| 47 | Kułakowskaa et al., 2017 [76] | 0 | 0 | 1 | 1 | 1 | 0 | 0 | 0 | 3 |
| 48 | Potemkowski and Jasinska, 2015 [77] | 0 | 0 | 1 | 1 | 1 | 0 | 0 | 1 | 4 |
| **Portugal** | | | | | | | | | | |
| 49 | De Sa et al., 2006) [79] | 0 | 0 | 1 | 1 | 1 | 1 | 1 | 1 | 6 |
| 50 | Figueiredo et al., 2015 [80] | 0 | 0 | 1 | 1 | 1 | 0 | 1 | 1 | 5 |
| 51 | Ruano et al., 2014 [82] | 0 | 0 | 1 | 1 | 1 | 0 | 1 | 0 | 4 |
| 52 | Branco et al., 2020 [78] | 0 | 0 | 1 | 1 | 1 | 0 | 1 | 1 | 5 |
| 53 | Lopes et al., 2020 [81] | 0 | 0 | 1 | 1 | 1 | 0 | 0 | 1 | 4 |
| **Romania** | | | | | | | | | | |
| 54 | Becus and Popoviciu, 1994 [83] | 0 | 0 | 1 | 0 | 1 | 0 | 0 | 0 | 2 |
| 55 | Cornea et al., 2016 [84] | 0 | 0 | 1 | 0 | 1 | 0 | 0 | 1 | 3 |
| **Serbia** | | | | | | | | | | |
| 56 | Toncev et al., 2011 [86] | 0 | 0 | 1 | 1 | 1 | 0 | 0 | 1 | 4 |
| 57 | Pekmezovic et al., 2019 [85] | 0 | 0 | 1 | 1 | 1 | 0 | 0 | 1 | 4 |
| **Spain** | | | | | | | | | | |
| 58 | Aladro et al., 2005 [87] | 0 | 0 | 1 | 1 | 1 | 0 | 1 | 1 | 5 |
| 59 | Benito-Leon et al., 1998 [88] | 0 | 0 | 1 | 1 | 1 | 0 | 1 | 1 | 5 |
| 60 | Bufill et al., 1995 [89] | 0 | 0 | 1 | 1 | 1 | 0 | 0 | 1 | 4 |
| 61 | Candeliere-Merlicco et al., 2016 [90] | 0 | 0 | 1 | 1 | 1 | 0 | 1 | 1 | 5 |
| 62 | Casquero et al., 2001 [91] | 0 | 0 | 1 | 1 | 1 | 0 | 1 | 1 | 5 |
| 63 | Hernandez, 2002 [93] | 0 | 0 | 1 | 1 | 1 | 0 | 0 | 1 | 4 |
| 64 | Izquierdo et al., 2015 [94] | 0 | 0 | 1 | 1 | 1 | 0 | 1 | 1 | 5 |
| 65 | Modrego and Pina, 2003 [96] | 0 | 0 | 1 | 1 | 1 | 0 | 1 | 1 | 5 |
| 66 | Modrego Pardo et al., 1997 [95] | 0 | 0 | 1 | 1 | 1 | 0 | 1 | 1 | 5 |
| 67 | Perez-Carmona et al., 2017 [18] | 1 | 0 | 1 | 1 | 0 | 0 | 0 | 0 | 3 |
| 68 | Pina et al., 1998 [98] | 0 | 0 | 1 | 1 | 1 | 0 | 1 | 1 | 5 |
| 69 | Tola et al., 1999 [99] | 0 | 0 | 1 | 1 | 1 | 0 | 1 | 1 | 5 |
| 70 | Perez-Carmona et al., 2019 [97] | 0 | 0 | 1 | 1 | 1 | 0 | 1 | 1 | 5 |
| 71 | Costa Arpin et al., 2020 [92] | 0 | 0 | 1 | 1 | 1 | 0 | 1 | 1 | 5 |
| **Sweden** | | | | | | | | | | |
| 72 | Bostrom et al., 2009 [100] | 0 | 0 | 1 | 1 | 0 | 0 | 1 | 1 | 4 |
| **Turkey** | | | | | | | | | | |
| 73 | Turk Boru et al., 2006 [104] | 1 | 1 | 1 | 1 | 1 | 1 | 0 | 1 | 7 |
| 74 | Çelik et al., 2011 [102] | 0 | 0 | 1 | 1 | 1 | 0 | 1 | 1 | 5 |
| 75 | Turk Boru et al., 2011 [105] | 1 | 1 | 1 | 1 | 1 | 1 | 1 | 1 | 8 |
| 76 | Akdemir et al., 2017 [101] | 0 | 0 | 1 | 1 | 1 | 0 | 0 | 1 | 4 |
| 77 | Turk Boru et al., 2018 [106] | 1 | 1 | 1 | 1 | 1 | 1 | 0 | 1 | 7 |
| 78 | Gokce et al., 2019 [103] | 1 | 1 | 1 | 1 | 1 | 1 | 0 | 1 | 7 |
| 79 | Turk Boru et al., 2020 [107] | 1 | 1 | 1 | 1 | 1 | 1 | 0 | 1 | 7 |
| **United Kingdom** | | | | | | | | | | |
| 80 | Ford et al., 1998 [108] | 0 | 0 | 1 | 1 | 1 | 0 | 0 | 1 | 4 |
| 81 | Ford et al., 2002 [109] | 0 | 0 | 1 | 1 | 1 | 0 | 1 | 1 | 5 |
| 82 | Fox et al., 2004 [110] | 0 | 0 | 1 | 1 | 1 | 0 | 1 | 1 | 5 |
| 83 | Gajofatto et al., 2013 [111] | 0 | 0 | 1 | 1 | 1 | 0 | 1 | 1 | 5 |
| 84 | Robertson et al., 1995 [112] |  |  |  |  |  |  |  |  |  |
| 85 | Simpson et al., 2015 [113] | 0 | 0 | 1 | 1 | 1 | 0 | 1 | 1 | 5 |
| 86 | Visser et al., 2012 [114] | 0 | 0 | 1 | 1 | 1 | 0 | 1 | 1 | 5 |
| **Australia** | | | | | | | | | | |
| 87 | Barnett et al., 2003 [19] | 0 | 0 | 1 | 1 | 1 | 0 | 1 | 1 | 5 |
| 88 | Ribbons et al., 2017 [20] | 0 | 0 | 1 | 1 | 1 | 0 | 1 | 1 | 5 |
| **Brazil** | | | | | | | | | | |
| 89 | Callegaro et al., 2001 [21] | 0 | 0 | 1 | 1 | 1 | 0 | 0 | 1 | 4 |
| 90 | Calmon et al., 2016 [22] | 0 | 0 | 1 | 1 | 1 | 0 | 1 | 1 | 5 |
| 91 | Negreiros et al., 2015 [24] | 0 | 0 | 1 | 1 | 1 | 0 | 0 | 1 | 4 |
| 92 | Lana-Peixoto et al., 2012 [23] | 0 | 0 | 1 | 1 | 1 | 0 | 0 | 1 | 4 |
| 93 | Ribeiro et al., 2011 [25] | 0 | 0 | 1 | 1 | 1 | 0 | 0 | 1 | 4 |
| 94 | Ribeiro et al., 2019 [26] | 0 | 0 | 1 | 1 | 1 | 0 | 0 | 1 | 4 |
| **Canada** | | | | | | | | | | |
| 95 | Sloka et al., 2005 [27] | 0 | 0 | 1 | 1 | 1 | 0 | 1 | 1 | 5 |
| 96 | Warren and Warren, 1992 [28] | 0 | 0 | 1 | 1 | 1 | 0 | 1 | 1 | 5 |
| 97 | Warren and Warren, 1993 [29] | 0 | 0 | 1 | 1 | 1 | 0 | 1 | 1 | 5 |

CA, critical appraisal; SPMS, secondary progressive multiple sclerosis.

**Table 2. Leave-one-out analysis result**

|  | Estimate | zval | pval | ci.lb | ci.ub | Q | Qp | tau2 | I2 | H2 |
| --- | --- | --- | --- | --- | --- | --- | --- | --- | --- | --- |
| 1 | 0.000226 | 20.809232 | 0.000000 | 0.000172 | 0.000287 | 10690.493001 | 0.000000 | 0.000048 | 98.923532 | 92.896385 |
| 2 | 0.000226 | 20.779875 | 0.000000 | 0.000172 | 0.000287 | 10691.038651 | 0.000000 | 0.000048 | 98.925041 | 93.026845 |
| 3 | 0.000226 | 20.820237 | 0.000000 | 0.000172 | 0.000287 | 10688.878326 | 0.000000 | 0.000048 | 98.918302 | 92.447278 |
| 4 | 0.000226 | 20.814409 | 0.000000 | 0.000172 | 0.000287 | 10689.380577 | 0.000000 | 0.000048 | 98.918928 | 92.500815 |
| 5 | 0.000228 | 21.063447 | 0.000000 | 0.000174 | 0.000288 | 10588.981474 | 0.000000 | 0.000047 | 98.888658 | 89.981339 |
| 6 | 0.000227 | 21.018791 | 0.000000 | 0.000174 | 0.000288 | 10440.625871 | 0.000000 | 0.000047 | 98.853569 | 87.227237 |
| 7 | 0.000228 | 21.217573 | 0.000000 | 0.000175 | 0.000289 | 10614.100639 | 0.000000 | 0.000046 | 98.886562 | 89.811877 |
| 8 | 0.000228 | 21.119989 | 0.000000 | 0.000174 | 0.000288 | 10426.159305 | 0.000000 | 0.000047 | 98.865530 | 88.146869 |
| 9 | 0.000229 | 21.343260 | 0.000000 | 0.000175 | 0.000289 | 6477.821032 | 0.000000 | 0.000046 | 98.651270 | 74.143829 |
| 10 | 0.000228 | 21.260296 | 0.000000 | 0.000175 | 0.000289 | 10611.524266 | 0.000000 | 0.000046 | 98.883490 | 89.564773 |
| 11 | 0.000225 | 20.723094 | 0.000000 | 0.000171 | 0.000286 | 10690.018222 | 0.000000 | 0.000048 | 98.929953 | 93.453824 |
| 12 | 0.000224 | 20.688589 | 0.000000 | 0.000170 | 0.000285 | 10687.841816 | 0.000000 | 0.000048 | 98.931256 | 93.567811 |
| 13 | 0.000225 | 20.701593 | 0.000000 | 0.000171 | 0.000286 | 10679.213166 | 0.000000 | 0.000048 | 98.919811 | 92.576403 |
| 14 | 0.000221 | 20.858619 | 0.000000 | 0.000168 | 0.000280 | 10675.248967 | 0.000000 | 0.000046 | 98.899298 | 90.851074 |
| 15 | 0.000221 | 20.813130 | 0.000000 | 0.000168 | 0.000281 | 10676.745898 | 0.000000 | 0.000047 | 98.905701 | 91.382678 |
| 16 | 0.000221 | 20.749075 | 0.000000 | 0.000168 | 0.000281 | 10647.298849 | 0.000000 | 0.000047 | 98.909199 | 91.675721 |
| 17 | 0.000224 | 20.655880 | 0.000000 | 0.000170 | 0.000285 | 10395.936214 | 0.000000 | 0.000048 | 98.863107 | 87.959051 |
| 18 | 0.000223 | 20.653001 | 0.000000 | 0.000169 | 0.000283 | 9901.613323 | 0.000000 | 0.000048 | 98.868719 | 88.395346 |
| 19 | 0.000222 | 20.682078 | 0.000000 | 0.000169 | 0.000282 | 10594.556698 | 0.000000 | 0.000047 | 98.916031 | 92.253551 |
| 20 | 0.000226 | 20.772811 | 0.000000 | 0.000172 | 0.000287 | 10691.061459 | 0.000000 | 0.000048 | 98.919139 | 92.518811 |
| 21 | 0.000227 | 21.051406 | 0.000000 | 0.000174 | 0.000288 | 10663.951699 | 0.000000 | 0.000047 | 98.903224 | 91.176289 |
| 22 | 0.000224 | 20.658868 | 0.000000 | 0.000170 | 0.000285 | 10651.748680 | 0.000000 | 0.000048 | 98.923330 | 92.879005 |
| 23 | 0.000226 | 20.767700 | 0.000000 | 0.000172 | 0.000287 | 10691.062176 | 0.000000 | 0.000048 | 98.918908 | 92.499059 |
| 24 | 0.000220 | 20.808473 | 0.000000 | 0.000168 | 0.000280 | 10538.408794 | 0.000000 | 0.000046 | 98.895697 | 90.554829 |
| 25 | 0.000218 | 21.175471 | 0.000000 | 0.000167 | 0.000276 | 10469.856799 | 0.000000 | 0.000044 | 98.844255 | 86.524299 |
| 26 | 0.000221 | 20.759411 | 0.000000 | 0.000168 | 0.000281 | 10596.404183 | 0.000000 | 0.000047 | 98.904798 | 91.307384 |
| 27 | 0.000222 | 20.693279 | 0.000000 | 0.000169 | 0.000282 | 10637.921444 | 0.000000 | 0.000047 | 98.917095 | 92.344170 |
| 28 | 0.000220 | 20.853217 | 0.000000 | 0.000168 | 0.000279 | 10331.402844 | 0.000000 | 0.000046 | 98.883990 | 89.604912 |
| 29 | 0.000220 | 20.836409 | 0.000000 | 0.000168 | 0.000280 | 10534.177575 | 0.000000 | 0.000046 | 98.891699 | 90.228188 |
| 30 | 0.000222 | 20.680864 | 0.000000 | 0.000169 | 0.000283 | 10643.225265 | 0.000000 | 0.000047 | 98.919537 | 92.552890 |
| 31 | 0.000220 | 20.814493 | 0.000000 | 0.000168 | 0.000280 | 10564.300265 | 0.000000 | 0.000046 | 98.895719 | 90.556623 |
| 32 | 0.000223 | 20.665020 | 0.000000 | 0.000170 | 0.000284 | 10679.981069 | 0.000000 | 0.000048 | 98.928819 | 93.354904 |
| 33 | 0.000221 | 20.706750 | 0.000000 | 0.000168 | 0.000282 | 10363.975235 | 0.000000 | 0.000047 | 98.902044 | 91.078291 |
| 34 | 0.000223 | 20.676706 | 0.000000 | 0.000169 | 0.000284 | 10681.728870 | 0.000000 | 0.000048 | 98.927234 | 93.216950 |
| 35 | 0.000226 | 20.834881 | 0.000000 | 0.000172 | 0.000287 | 10684.215913 | 0.000000 | 0.000048 | 98.909843 | 91.729883 |
| 36 | 0.000224 | 20.669178 | 0.000000 | 0.000170 | 0.000285 | 10672.728756 | 0.000000 | 0.000048 | 98.927092 | 93.204653 |
| 37 | 0.000225 | 20.684522 | 0.000000 | 0.000171 | 0.000286 | 10676.008198 | 0.000000 | 0.000048 | 98.924384 | 92.969939 |
| 38 | 0.000225 | 20.726480 | 0.000000 | 0.000171 | 0.000286 | 10690.355214 | 0.000000 | 0.000048 | 98.930632 | 93.513188 |
| 39 | 0.000225 | 20.726480 | 0.000000 | 0.000171 | 0.000286 | 10690.355214 | 0.000000 | 0.000048 | 98.930632 | 93.513188 |
| 40 | 0.000222 | 20.672397 | 0.000000 | 0.000169 | 0.000283 | 10537.327947 | 0.000000 | 0.000047 | 98.913904 | 92.072862 |
| 41 | 0.000224 | 20.670579 | 0.000000 | 0.000170 | 0.000285 | 10684.032473 | 0.000000 | 0.000048 | 98.930756 | 93.524055 |
| 42 | 0.000228 | 21.047100 | 0.000000 | 0.000174 | 0.000288 | 10558.442205 | 0.000000 | 0.000047 | 98.882323 | 89.471293 |
| 43 | 0.000224 | 20.676879 | 0.000000 | 0.000170 | 0.000285 | 10671.385793 | 0.000000 | 0.000048 | 98.924333 | 92.965616 |
| 44 | 0.000225 | 20.708023 | 0.000000 | 0.000171 | 0.000286 | 10682.280017 | 0.000000 | 0.000048 | 98.920316 | 92.619679 |
| 45 | 0.000225 | 20.684221 | 0.000000 | 0.000171 | 0.000286 | 10676.848875 | 0.000000 | 0.000048 | 98.925024 | 93.025344 |
| 46 | 0.000224 | 20.663049 | 0.000000 | 0.000170 | 0.000285 | 10663.836991 | 0.000000 | 0.000048 | 98.925553 | 93.071175 |
| 47 | 0.000224 | 20.651556 | 0.000000 | 0.000170 | 0.000285 | 10646.941976 | 0.000000 | 0.000048 | 98.924716 | 92.998657 |
| 48 | 0.000224 | 20.708118 | 0.000000 | 0.000170 | 0.000285 | 10689.495661 | 0.000000 | 0.000048 | 98.930876 | 93.534562 |
| 49 | 0.000225 | 20.716062 | 0.000000 | 0.000171 | 0.000286 | 10679.634167 | 0.000000 | 0.000048 | 98.909413 | 91.693738 |
| 50 | 0.000226 | 20.812481 | 0.000000 | 0.000172 | 0.000287 | 10689.531381 | 0.000000 | 0.000048 | 98.919133 | 92.518351 |
| 51 | 0.000222 | 20.677663 | 0.000000 | 0.000169 | 0.000283 | 10634.310915 | 0.000000 | 0.000047 | 98.919349 | 92.536828 |
| 52 | 0.000222 | 20.698253 | 0.000000 | 0.000168 | 0.000282 | 10589.559060 | 0.000000 | 0.000047 | 98.913396 | 92.029825 |
| 53 | 0.000224 | 20.658716 | 0.000000 | 0.000170 | 0.000285 | 10573.026651 | 0.000000 | 0.000048 | 98.901877 | 91.064464 |
| 54 | 0.000224 | 20.649031 | 0.000000 | 0.000170 | 0.000285 | 10514.384103 | 0.000000 | 0.000048 | 98.901265 | 91.013749 |
| 55 | 0.000224 | 20.660901 | 0.000000 | 0.000170 | 0.000285 | 10581.410375 | 0.000000 | 0.000048 | 98.901816 | 91.059411 |
| 56 | 0.000224 | 20.653883 | 0.000000 | 0.000170 | 0.000285 | 10556.560669 | 0.000000 | 0.000048 | 98.903317 | 91.184081 |
| 57 | 0.000223 | 20.650463 | 0.000000 | 0.000169 | 0.000284 | 10642.227534 | 0.000000 | 0.000048 | 98.924723 | 92.999250 |
| 58 | 0.000225 | 20.756181 | 0.000000 | 0.000171 | 0.000287 | 10690.858311 | 0.000000 | 0.000048 | 98.923282 | 92.874797 |
| 59 | 0.000226 | 20.805701 | 0.000000 | 0.000172 | 0.000287 | 10690.865448 | 0.000000 | 0.000048 | 98.925651 | 93.079656 |
| 60 | 0.000227 | 20.923828 | 0.000000 | 0.000173 | 0.000288 | 10647.233100 | 0.000000 | 0.000047 | 98.896499 | 90.620633 |
| 61 | 0.000227 | 21.018893 | 0.000000 | 0.000174 | 0.000288 | 10675.227912 | 0.000000 | 0.000047 | 98.907554 | 91.537683 |
| 62 | 0.000226 | 20.766686 | 0.000000 | 0.000172 | 0.000287 | 10691.055300 | 0.000000 | 0.000048 | 98.922661 | 92.821255 |
| 63 | 0.000223 | 20.645059 | 0.000000 | 0.000169 | 0.000284 | 10290.729573 | 0.000000 | 0.000048 | 98.887950 | 89.923992 |
| 64 | 0.000224 | 20.682345 | 0.000000 | 0.000171 | 0.000286 | 10677.345933 | 0.000000 | 0.000048 | 98.925903 | 93.101473 |
| 65 | 0.000225 | 20.738803 | 0.000000 | 0.000171 | 0.000286 | 10690.584092 | 0.000000 | 0.000048 | 98.929029 | 93.373236 |
| 66 | 0.000224 | 20.668326 | 0.000000 | 0.000170 | 0.000285 | 10681.786873 | 0.000000 | 0.000048 | 98.930446 | 93.496956 |
| 67 | 0.000227 | 20.951844 | 0.000000 | 0.000173 | 0.000288 | 10677.674556 | 0.000000 | 0.000047 | 98.911394 | 91.860628 |
| 68 | 0.000226 | 20.830670 | 0.000000 | 0.000172 | 0.000287 | 10690.341771 | 0.000000 | 0.000048 | 98.923727 | 92.913264 |
| 69 | 0.000225 | 20.724129 | 0.000000 | 0.000171 | 0.000286 | 10689.325375 | 0.000000 | 0.000048 | 98.927568 | 93.246026 |
| 70 | 0.000225 | 20.751329 | 0.000000 | 0.000171 | 0.000286 | 10690.891334 | 0.000000 | 0.000048 | 98.928768 | 93.350490 |
| 71 | 0.000225 | 20.750522 | 0.000000 | 0.000171 | 0.000286 | 10690.866766 | 0.000000 | 0.000048 | 98.928450 | 93.322773 |
| 72 | 0.000225 | 20.736029 | 0.000000 | 0.000171 | 0.000286 | 10690.185505 | 0.000000 | 0.000048 | 98.927175 | 93.211883 |
| 73 | 0.000226 | 20.824033 | 0.000000 | 0.000172 | 0.000287 | 10690.601728 | 0.000000 | 0.000048 | 98.924556 | 92.984876 |
| 74 | 0.000227 | 21.027307 | 0.000000 | 0.000174 | 0.000288 | 10673.647975 | 0.000000 | 0.000047 | 98.906686 | 91.465034 |
| 75 | 0.000225 | 20.745230 | 0.000000 | 0.000171 | 0.000286 | 10690.792433 | 0.000000 | 0.000048 | 98.929323 | 93.398864 |
| 76 | 0.000223 | 20.663776 | 0.000000 | 0.000170 | 0.000284 | 10680.590253 | 0.000000 | 0.000048 | 98.929584 | 93.421640 |
| 77 | 0.000226 | 20.878077 | 0.000000 | 0.000173 | 0.000287 | 10688.334621 | 0.000000 | 0.000047 | 98.919783 | 92.574016 |
| 78 | 0.000220 | 20.851838 | 0.000000 | 0.000168 | 0.000279 | 10397.631402 | 0.000000 | 0.000046 | 98.885858 | 89.755150 |
| 79 | 0.000228 | 21.112331 | 0.000000 | 0.000174 | 0.000288 | 10005.209220 | 0.000000 | 0.000047 | 98.808694 | 83.941477 |
| 80 | 0.000222 | 20.769193 | 0.000000 | 0.000169 | 0.000282 | 10685.136553 | 0.000000 | 0.000047 | 98.917804 | 92.404682 |
| 81 | 0.000222 | 20.769193 | 0.000000 | 0.000169 | 0.000282 | 10685.136553 | 0.000000 | 0.000047 | 98.917804 | 92.404682 |
| 82 | 0.000223 | 20.675687 | 0.000000 | 0.000170 | 0.000284 | 10683.324550 | 0.000000 | 0.000048 | 98.928415 | 93.319710 |
| 83 | 0.000225 | 20.719014 | 0.000000 | 0.000171 | 0.000286 | 10689.986201 | 0.000000 | 0.000048 | 98.930563 | 93.507150 |
| 84 | 0.000225 | 20.811232 | 0.000000 | 0.000172 | 0.000286 | 10691.046006 | 0.000000 | 0.000048 | 98.926878 | 93.186069 |
| 85 | 0.000226 | 20.832007 | 0.000000 | 0.000172 | 0.000287 | 10690.910356 | 0.000000 | 0.000048 | 98.925523 | 93.068575 |
| 86 | 0.000225 | 20.748739 | 0.000000 | 0.000171 | 0.000286 | 10690.846274 | 0.000000 | 0.000048 | 98.929770 | 93.437847 |
| 87 | 0.000225 | 20.734297 | 0.000000 | 0.000171 | 0.000286 | 10690.575527 | 0.000000 | 0.000048 | 98.930330 | 93.486801 |
| 88 | 0.000225 | 20.799257 | 0.000000 | 0.000172 | 0.000286 | 10691.051262 | 0.000000 | 0.000048 | 98.927278 | 93.220834 |
| 89 | 0.000223 | 20.646732 | 0.000000 | 0.000170 | 0.000284 | 10566.727059 | 0.000000 | 0.000048 | 98.913694 | 92.055092 |
| 90 | 0.000223 | 20.645553 | 0.000000 | 0.000169 | 0.000284 | 10537.300495 | 0.000000 | 0.000048 | 98.912600 | 91.962440 |
| 91 | 0.000223 | 20.648182 | 0.000000 | 0.000169 | 0.000284 | 10607.942398 | 0.000000 | 0.000048 | 98.921267 | 92.701373 |
| 92 | 0.000224 | 20.672204 | 0.000000 | 0.000170 | 0.000285 | 10667.403629 | 0.000000 | 0.000048 | 98.924049 | 92.940994 |
| 93 | 0.000221 | 20.805069 | 0.000000 | 0.000168 | 0.000280 | 10614.174056 | 0.000000 | 0.000047 | 98.899008 | 90.827171 |
| 94 | 0.000220 | 20.838842 | 0.000000 | 0.000168 | 0.000280 | 10600.765719 | 0.000000 | 0.000046 | 98.893625 | 90.385239 |
| 95 | 0.000219 | 21.046019 | 0.000000 | 0.000167 | 0.000277 | 10297.637322 | 0.000000 | 0.000045 | 98.858319 | 87.590112 |

**Figure 1. SPMS prevalence (per 100,000) pattern in Australia**


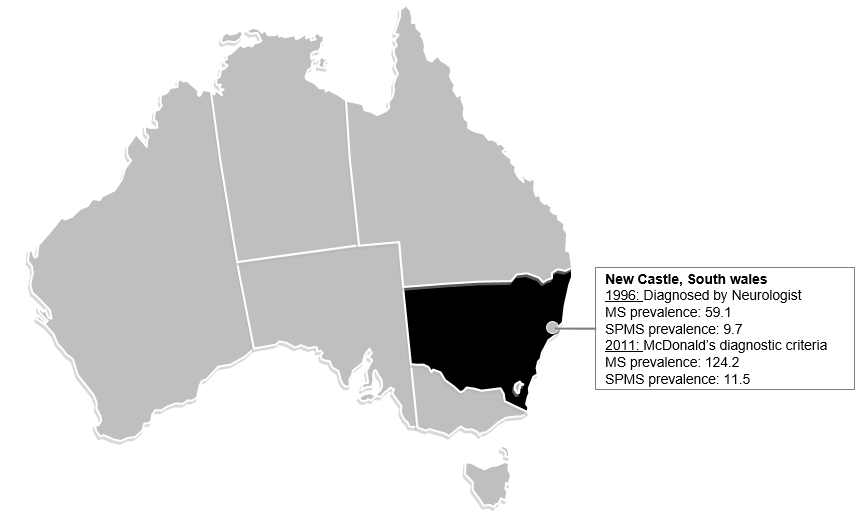


MS, multiple sclerosis; SPMS, secondary progressive multiple sclerosis.

Figure 2. SPMS prevalence (per 100,000) pattern in Hungary


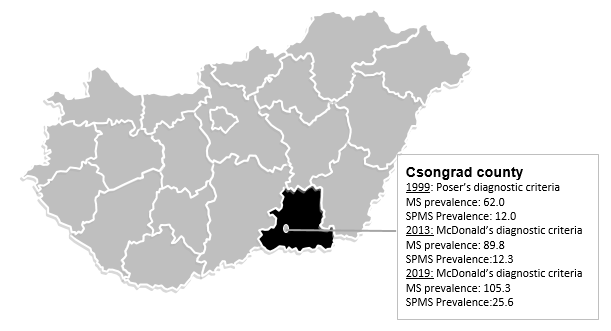


MS, multiple sclerosis; SPMS, secondary progressive multiple sclerosis.

Figure 3. SPMS prevalence (per 100,000) pattern in Ireland


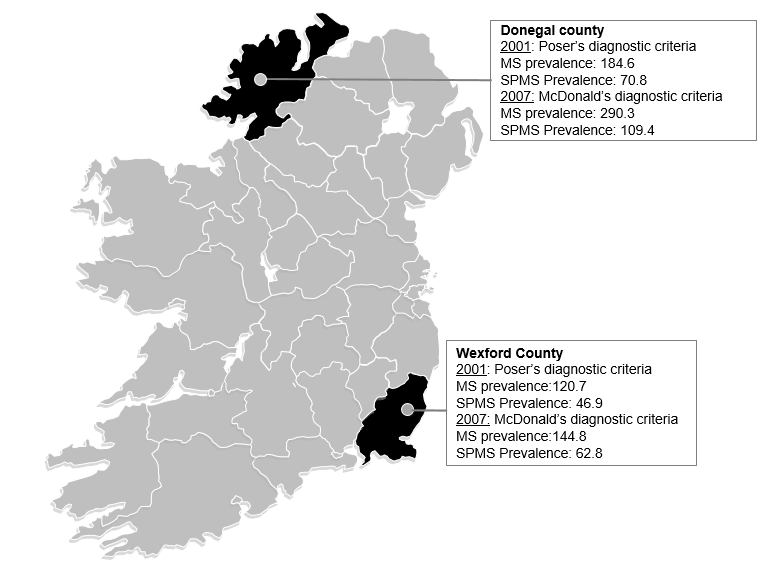


MS, multiple sclerosis; SPMS, secondary progressive multiple sclerosis.

Figure 4. SPMS prevalence (per 100,000) pattern in Italy


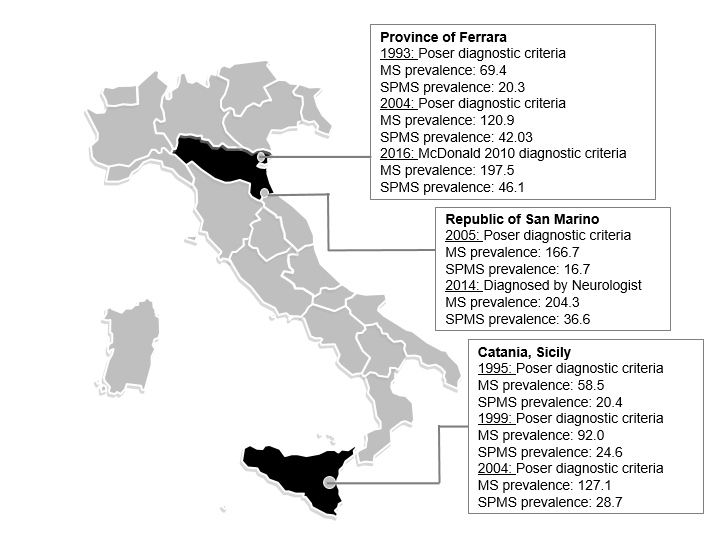


MS, multiple sclerosis; SPMS, secondary progressive multiple sclerosis.

Figure 5. SPMS prevalence (per 100,000) pattern in Poland


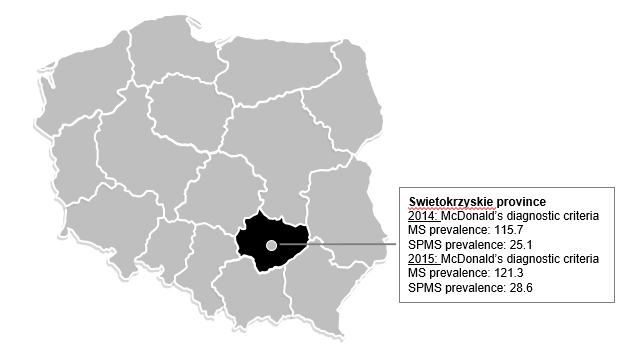


MS, multiple sclerosis; SPMS, secondary progressive multiple sclerosis.

**Figure 6. SPMS prevalence (per 100,000) pattern in the United Kingdom**
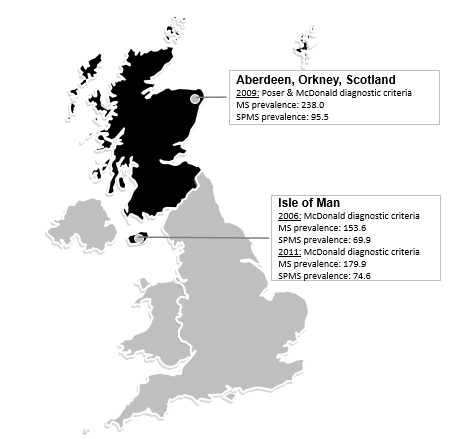

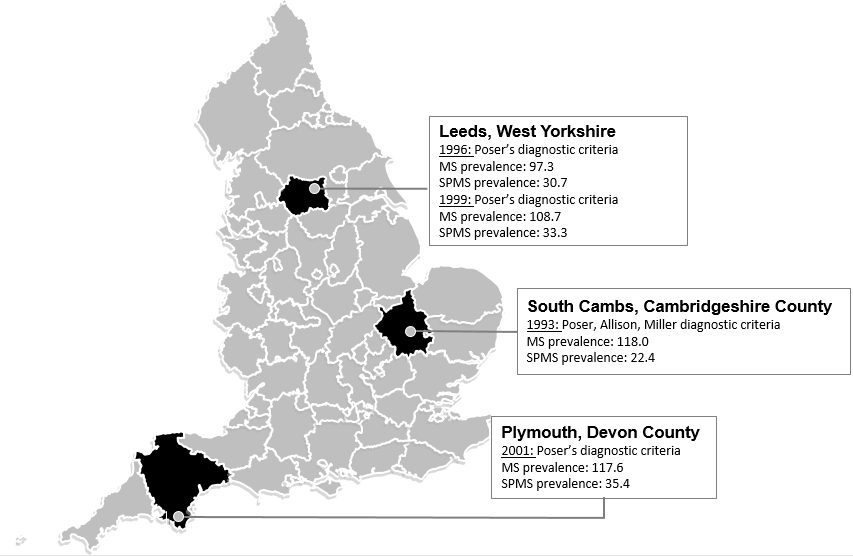


MS, multiple sclerosis; SPMS, secondary progressive multiple sclerosis.

**Figure 7. Funnel plot**


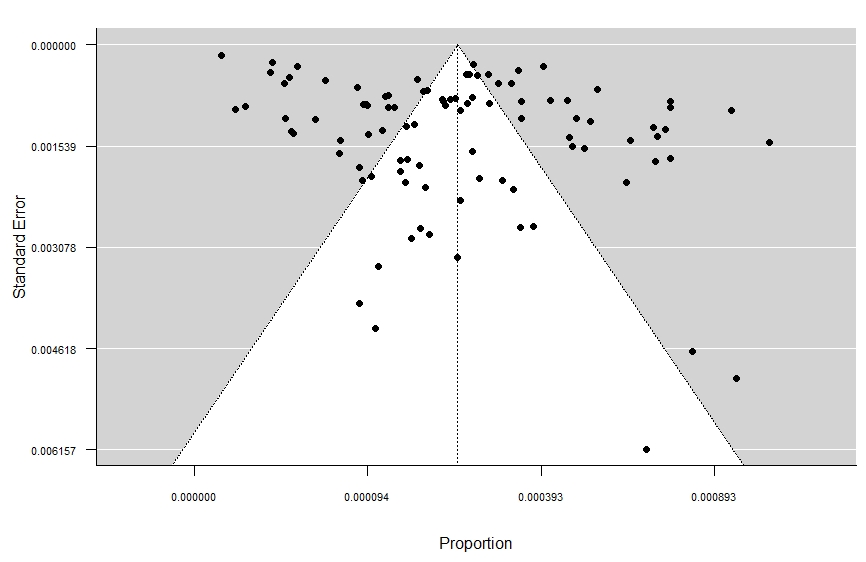


**Figure 8. Baujat plot**


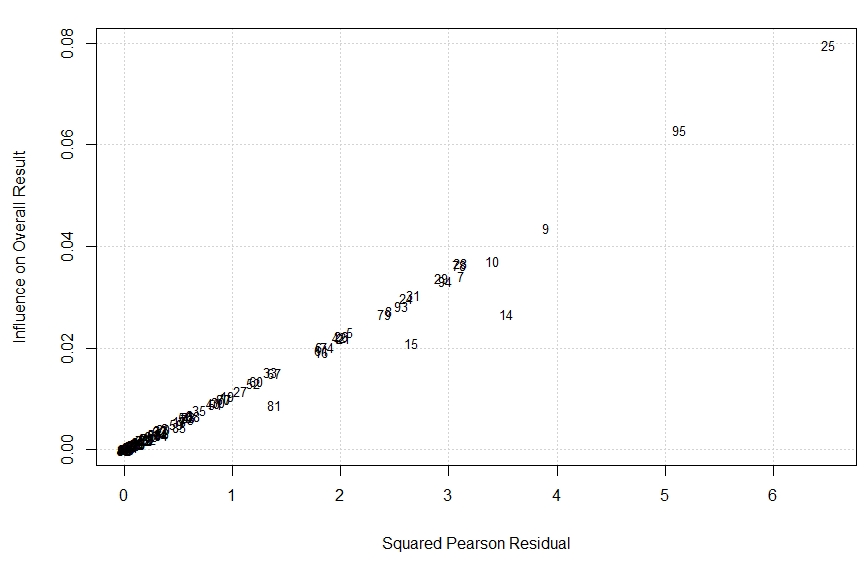


**Figure 9. Studentized residuals test**


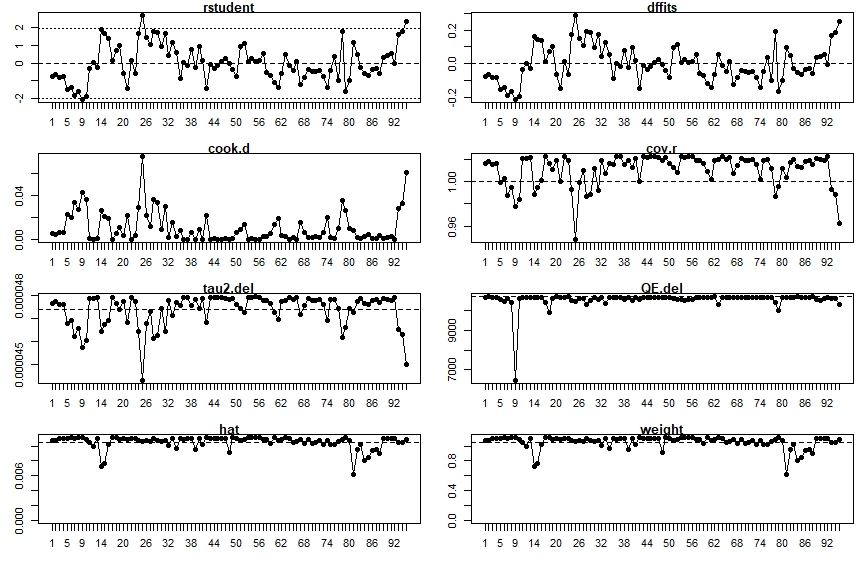


**Figure 10. Scatter plot of moderator region**


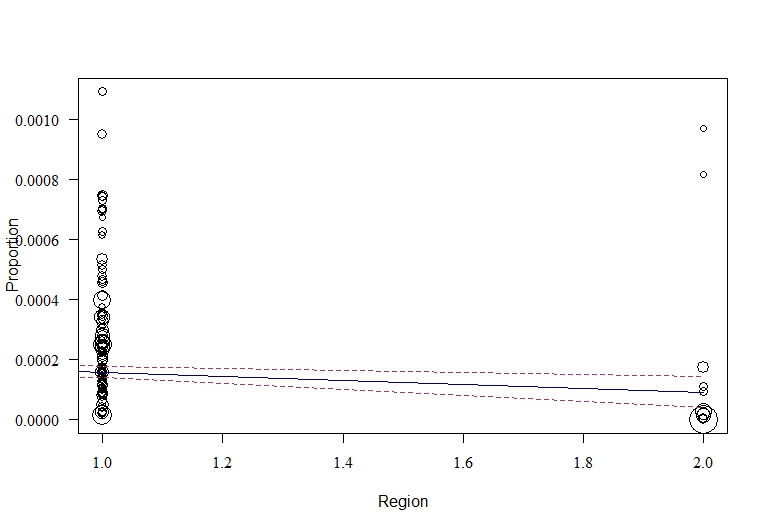


**Figure 11. Scatter plot of moderator introduction of oral DMT**


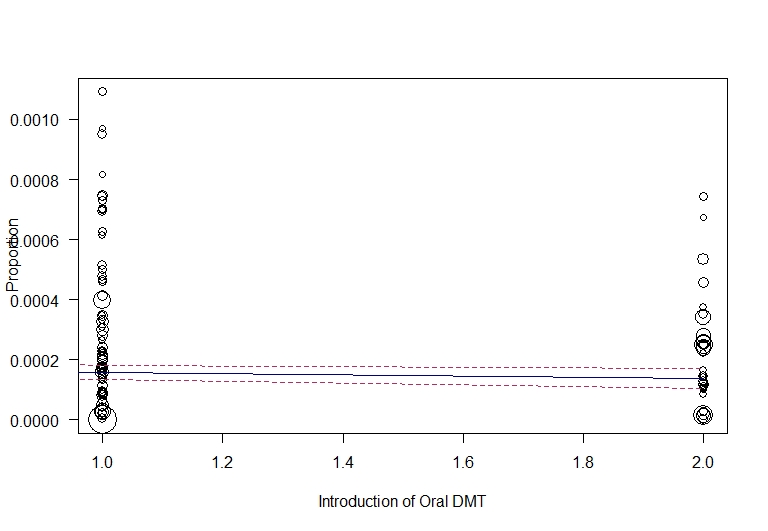


**Figure 12. Scatter plot of moderator sample size**


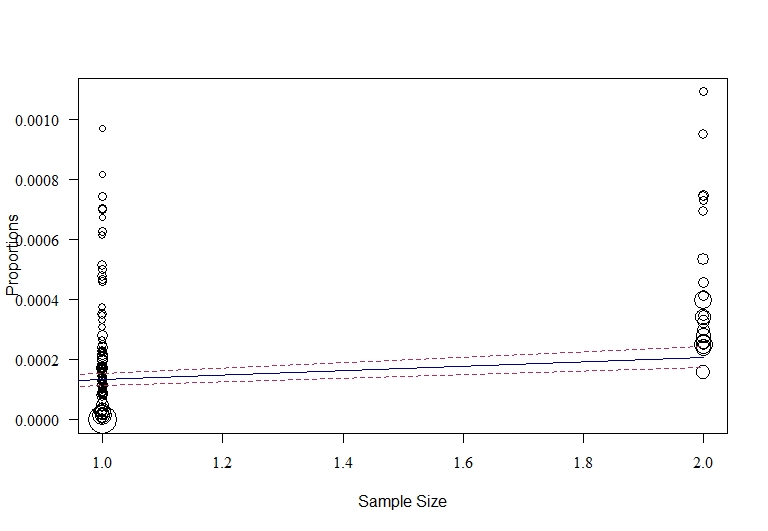


**Figure 13. Scatter plot of moderator diagnostic criteria**


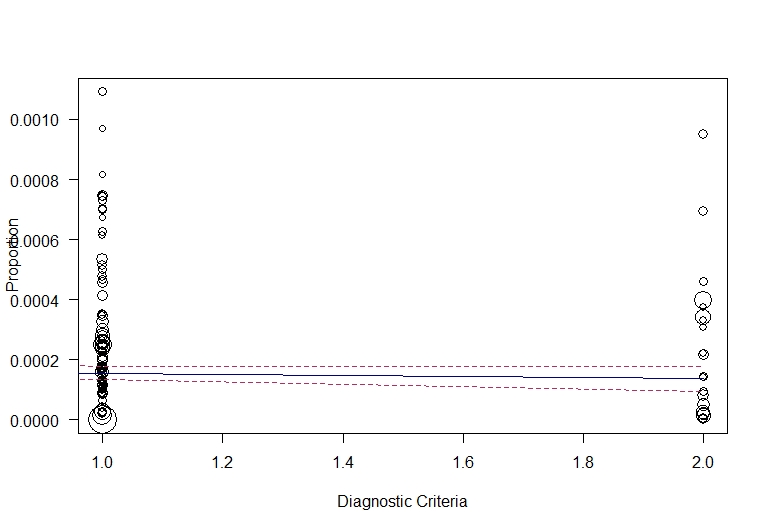

Supplement: Supplementary file 1 — Additional file 1: Table 1. Loney quality assessment of SPMS prevalence studies. Table 2. Leave-one-out analysis result. Figure 1. SPMS prevalence (per 100,000) pattern in Australia. Figure 2. SPMS prevalence (per 100,000) pattern in Hungary. Figure 3. SPMS prevalence (per 100,000) pattern in Ireland. Figure 4. SPMS prevalence (per 100,000) pattern in Italy. Figure 5. SPMS prevalence (per 100,000) pattern in Poland. Figure 6. SPMS prevalence (per 100,000) pattern in the United Kingdom. Figure 7. Funnel plot. Figure 8. Baujat plot. Figure 9. Studentized residuals test. Figure 10. Scatter plot of moderator region. Figure 11. Scatter plot of moderator introduction of oral DMT. Figure 12. Scatter plot of moderator sample size. Figure 13. Scatter plot of moderator diagnostic criteria. [file 12883_2022_2820_MOESM1_ESM.docx]
